# Supplementary material for: Protocol for the Development and Analysis of the Oxford and Reading Cognitive Comorbidity, Frailty and Ageing Research Database-Electronic Patient Records (ORCHARD-EPR)
Source: BMJ Open. 2024 May 30;14(5):e085126. doi: 10.1136/bmjopen-2024-085126 (PMC11141189; doi:10.1136/bmjopen-2024-085126)

Protocol for the Development and Analysis of the Oxford and Reading Cognitive Comorbidity, Frailty and Ageing Research Database - Electronic Patient Records (ORCHARD-EPR).

Emily Boucher,[a] Aimee Jell,[b] Sudhir Singh,[c] Jim Davies,[d] Tanya Smith,[e,f] Adam Pill,[e] Kinga Varnai,[g] Kerrie Woods,[g] David Walliker,[g] Aubretia McColl,[h] Sasha Shepperd,[i] Sarah T Pendlebury[a,c,j]

[a] Wolfson Centre for Prevention of Stroke and Dementia, Wolfson Building, Nuffield Department of Clinical Neurosciences, University of Oxford, UK

[b]Informatics Department, Oxford University Hospitals NHS Foundation Trust, UK

[c]Departments of Acute General (Internal) Medicine and Geratology, Oxford University Hospitals NHS Foundation Trust, UK

[d]Department of Computer Science, University of Oxford, UK

[e]Research Informatics Team, Research and Development Department, Oxford Health NHS Foundation Trust

[f]NIHR Oxford Health Biomedical Research Centre

[g]Research and Development Clinical Informatics, Oxford University Hospitals NHS Foundation Trust, UK

[h]Departments of Acute and Elderly Care Medicine, Royal Berkshire Hospitals NHS Foundation Trust

[i]Nuffield Department of Population Health, University of Oxford, UK

[j]NIHR Biomedical Research Centre, Oxford University Hospitals NHS Foundation Trust, UK

Address correspondence to: Professor Sarah Pendlebury, Wolfson Centre for Prevention of Stroke and Dementia, Wolfson Building, John Radcliffe Hospital, Oxford OX3 9DU

Email: sarah.pendlebury@ndcn.ox.ac.uk

Supplementary material

Supplemental Methods

ORCHARD-EPR Inclusion Criteria

Patients with unplanned admission or Same Day Emergency Care-SDEC attendance

All OUHFT in-patients with unplanned admission and acute ambulatory care (Same Day Emergency Care-SDEC) patients aged $\geq$65 years are included in the database with the relevant datafields extracted from the EPR by the OUHFT Information analysts. Similar data are automatically extracted for in-patients and SDEC patients aged <65 years with a completed cognitive screen indicating clinician concern about possible cognitive frailty (eg because of Parkinson's disease, stroke, multiple sclerosis, alcohol excess etc). Pseudonymised data from previous relevant OUHFT-approved audits conducted prior to the implementation of EPR are also included, for example to establish the prevalence of delirium and cognitive impairment using different cognitive tests in consecutive admissions to acute general medicine.[1,2]

Out-patients

In addition, relevant OUHFT-approved audit data may be included on out-patients (including patients aged<65 years) deemed to be at-risk of cognitive or physical frailty as indicated by referral to vascular specialty clinics (eg TIA/minor stroke, peripheral vascular disease), memory clinics or geriatric medicine clinics where multimorbidity (the presence of two or more long-term conditions) is common. Manual extraction of audit data by the usual care team is necessary since at present, EPR recording of out-patient assessments is generally in the form of free text powernotes rather than structured data.

EPR Powerforms

EPR powerforms are bespoke electronic proformas "built" by the OUHFT EPR team and designed to capture structured clinical data. The cognitive screening form is an example of a powerform (see supplemental figure). The data entered into such a powerform can be easily extracted and analysed including all the individual powerform items (eg the individual AMTS questions). The structured format of powerforms also allows the information contained within to be displayed automatically in different locations in EPR. For example, the cognitive screening powerform results populate the observations tab and the "cognitive assessments" tab where all the cognitive screening results over time and across different encounters are shown allowing clinicians to see the cognitive trajectory. This tab also displays the clinical frailty score assuming the relevant powerform has been completed, so that the cognitive

assessments are put in the context of a global frailty assessment. The structured powerform data can also be included in algorithms for example, the AMTS score or untestability is included in the delirium susceptibility score algorithm which is calculated automatically within EPR and displayed in real time.[3]

EPR Powernotes

EPR powernotes are created by clinicians as electronic free text entries and are therefore entirely unstructured. Such free text data cannot be easily extracted or analysed and require the use of natural language processing algorithms. Information entered into a free text powernote, for example the AMTS score or documentation of delirium diagnosis, cannot therefore be automatically displayed in other parts or EPR or used in algorithms and quickly becomes “lost” amongst the mass of other entries.

References

1. Pendlebury ST, Lovett N, Smith S, Dutta N, Bendon C, Lloyd-Lavery A, et al. Observational, longitudinal study of delirium in consecutive unselected acute medical admissions: age-specific rates and associated factors, mortality and re-admission. *BMJ Open* 2015;5(11):e007808-e007808.

2. Pendlebury ST, Klaus SP, Mather M, de Brito M, Wharton RM. Routine cognitive screening in older patients admitted to acute medicine: abbreviated mental test score (AMTS) and subjective memory complaint versus Montreal Cognitive Assessment and IQCODE. *Age Ageing* 2015;44:1000-1005.

3. Pendlebury ST, Lovett NG, Smith SC, Wharton R, Rothwell PM. Delirium risk stratification in consecutive unselected admissions to acute medicine: validation of a susceptibility score based on factors identified externally in pooled data for use at entry to the acute care pathway. *Age Ageing* 2017;46:226-231.

Supplemental Figure

Supplementary Figure. The OUHFT cognitive screening EPR powerform including the 10-point AMTS, a brief screen for cognitive problems with a drop down list for reason for lack of completion (e.g. patient too unwell, aphasia); recording of formal dementia diagnosis (recorded as yes/no/uncertain); and recording of delirium diagnosis (recorded as yes/no/uncertain) informed by the CAM.

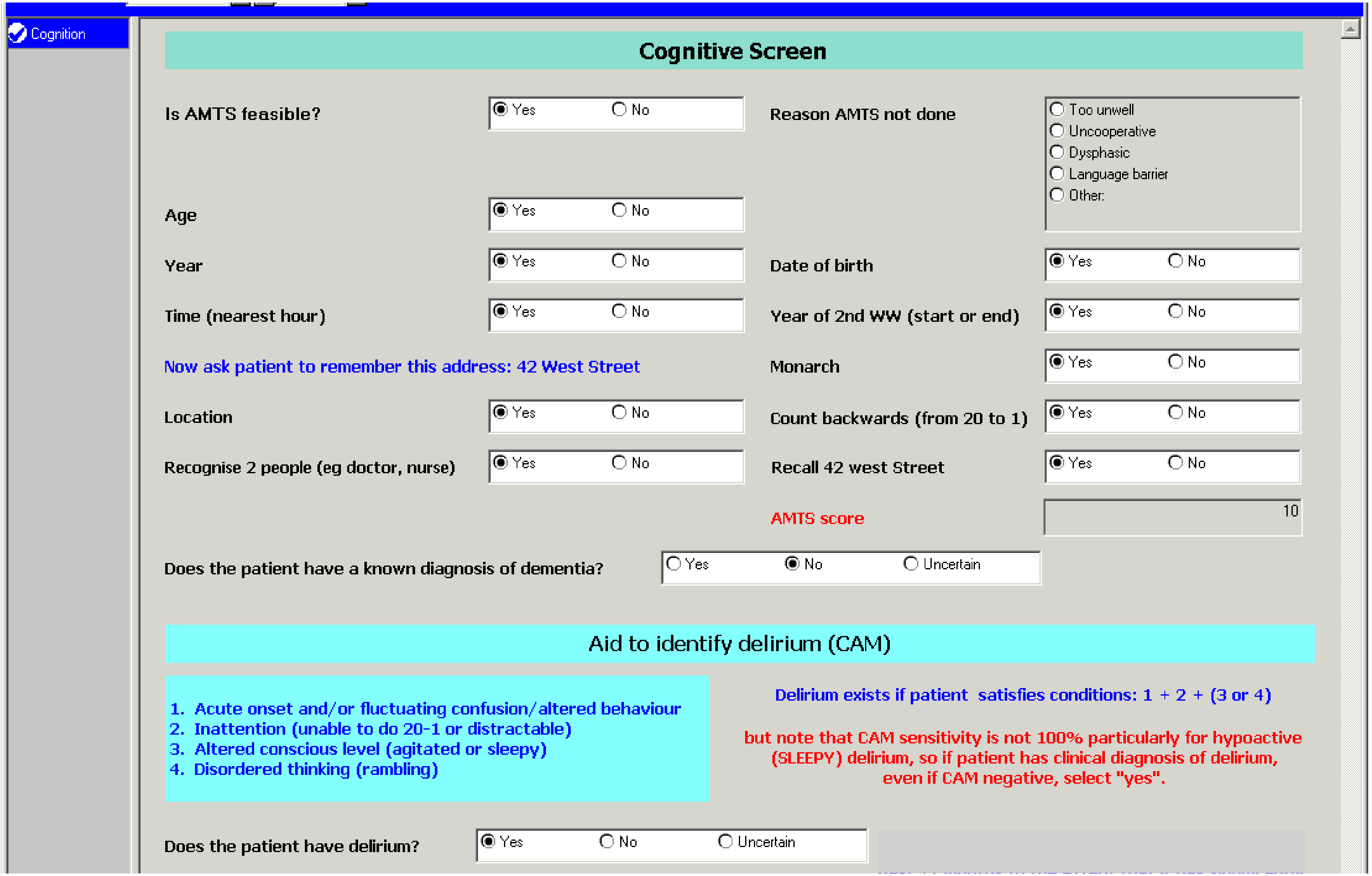

Supplement: Supplementary data [file bmjopen-2024-085126supp001.pdf]
