# Supplementary material for: Protocol for the Development and Analysis of the Oxford and Reading Cognitive Comorbidity, Frailty and Ageing Research Database-Electronic Patient Records (ORCHARD-EPR)
Source: BMJ Open. 2024 May 30;14(5):e085126. doi: 10.1136/bmjopen-2024-085126 (PMC11141189; doi:10.1136/bmjopen-2024-085126)
Supplement: Author's manuscript [file bmjopen-2024-085126.draft_revisions.pdf]

BMJ Open is committed to open peer review. As part of this commitment we make the peer review history of every article we publish publicly available.

When an article is published we post the peer reviewers' comments and the authors' responses online. We also post the versions of the paper that were used during peer review. These are the versions that the peer review comments apply to.

The versions of the paper that follow are the versions that were submitted during the peer review process. They are not the versions of record or the final published versions. They should not be cited or distributed as the published version of this manuscript.

BMJ Open is an open access journal and the full, final, typeset and author-corrected version of record of the manuscript is available on our site with no access controls, subscription charges or pay-per-view fees (http://bmjopen.bmj.com).

If you have any questions on BMJ Open's open peer review process please email info.bmjopen@bmj.com

BMJ Open

The Oxford and Reading Cognitive Comorbidity, Frailty and Ageing Research Database - Electronic Patient Records (ORCHARD-EPR): protocol

Journal:	*BMJ Open*
Manuscript ID	bmjopen-2024-085126
Article Type:	Protocol
Date Submitted by the Author:	13-Feb-2024

Complete List of Authors:	Boucher, Emily; University of Oxford Wolfson Centre for Prevention of Stroke and Dementia<br>Jell, Aimee; Oxford University Hospitals NHS Foundation Trust, Informatics Department<br>Singh, Sudhir; Oxford University Hospitals NHS Foundation Trust, Departments of Acute General (Internal) Medicine and Geratology<br>Davies, Jim; University of Oxford, Department of Computer Science<br>Smith, Tanya; Oxford Health NHS Foundation Trust, Research Informatics Team, Research and Development Department; NIHR Oxford Health Biomedical Research Centre<br>Pill, Adam; Oxford Health NHS Foundation Trust, Research Informatics Team, Research and Development Department<br>Varnai, Kinga; Oxford University Hospitals NHS Foundation Trust, Research and Development Clinical Informatics<br>Woods, Kerrie; Oxford University Hospitals NHS Foundation Trust, Research and Development Clinical Informatics<br>Walliker, David; Oxford University Hospitals NHS Foundation Trust, Research and Development Clinical Informatics<br>McColl, Aubretia; Royal Berkshire NHS Foundation Trust, Departments of Acute and Elderly Care Medicine<br>Shepperd, S; University of Oxford, Nuffield Department of Population Health<br>Pendlebury, Sarah; University of Oxford Wolfson Centre for Prevention of Stroke and Dementia, Nuffield Department of Clinical Neurosciences; Oxford University Hospitals NHS Foundation Trust, Departments of Acute General (Internal) Medicine and Geratology and NIHR Oxford Biomedical Research Centre

Keywords:	Electronic Health Records, Hospitals, Frailty, Dementia, Decision Making

SCHOLARONE™
Manuscripts

The Oxford and Reading Cognitive Comorbidity, Frailty and Ageing Research Database - Electronic Patient Records (ORCHARD-EPR): protocol

Emily Boucher,[a] Aimee Jell,[b] Sudhir Singh,[c] Jim Davies,[d] Tanya Smith,[e,f] Adam Pill,[e] Kinga Varnai,[g] Kerrie Woods,[g] David Walliker,[g] Aubretia McColl,[h] Sasha Shepperd,[i] Sarah T Pendlebury[a,c,j]

[a] Wolfson Centre for Prevention of Stroke and Dementia, Wolfson Building, Nuffield Department of Clinical Neurosciences, University of Oxford, UK

[b]Informatics Department, Oxford University Hospitals NHS Foundation Trust, UK

[c]Departments of Acute General (Internal) Medicine and Geratology, Oxford University Hospitals NHS Foundation Trust, UK

[d]Department of Computer Science, University of Oxford, UK

[e]Research Informatics Team, Research and Development Department, Oxford Health NHS Foundation Trust

[f]NIHR Oxford Health Biomedical Research Centre

[g]Research and Development Clinical Informatics, Oxford University Hospitals NHS Foundation Trust, UK

[h]Departments of Acute and Elderly Care Medicine, Royal Berkshire Hospitals NHS Foundation Trust

[i]Nuffield Department of Population Health, University of Oxford, UK

[j]NIHR Biomedical Research Centre, Oxford University Hospitals NHS Foundation Trust, UK

Address correspondence to: Professor Sarah Pendlebury, Wolfson Centre for Prevention of Stroke and Dementia, Wolfson Building, John Radcliffe Hospital, Oxford OX3 9DU

Email: sarah.pendlebury@ndcn.ox.ac.uk

Telephone: +44 1865 231603

For peer review only

Abstract (Max 300 words)

Background

Hospital electronic patient records (EPRs) offer the opportunity to exploit large scale routinely acquired data at relatively low cost and without selection. EPRs provide considerably richer data, and in real-time, than retrospective administrative datasets in which clinical complexity is often poorly captured. With population ageing, a wide range of hospital specialties now manage older people with multimorbidity, frailty and associated poor outcomes. We therefore set-up the Oxford and Reading Cognitive Comorbidity, Frailty and Ageing Research Database-Electronic Patient Records (ORCHARD-EPR) to facilitate clinically meaningful research in older hospital patients including algorithm development, and to aid medical decision making, implementation of guidelines, and inform policy.

Methods and analysis

ORCHARD-EPR uses routinely-acquired individual patient data on all patients aged ≥65 years with unplanned admission or Same Day Emergency Care unit attendance at four acute general hospitals serving a population of >800,000 (Oxfordshire, UK) with planned extension to the neighbouring Berkshire regional hospitals (>1,000,000). Datafields include diagnosis, comorbidities, nursing risk assessments, frailty, observations, illness acuity, laboratory tests and brain scan images. Importantly, ORCHARD-EPR contains the results from mandatory hospital-wide cognitive screening (≥70 years) comprising the 10-point Abbreviated-Mental-Test and dementia and delirium diagnosis (Confusion Assessment Method-CAM). Outcomes include length of stay, delayed transfers of care, discharge destination, readmissions, and death. The rich multimodal data are further enhanced by linkage to secondary care electronic mental health records. Selection of appropriate subgroups or linkage to existing cohorts allows disease specific studies.Over 200,000 patient episodes are included to date with data collection ongoing of which 129,248 are admissions with a LOS ≥1 day in 64,641 unique patients.

Ethics and dissemination

ORCHARD-EPR is approved by the South Central Oxford C Research Ethics Committee (ref: 23/SC/0258). Results will be widely disseminated through peer-reviewed publications and presentations at conferences, and regional meetings to improve hospital data quality and clinical services.

Keywords

Three to ten keywords representing the main content of the article.

Electronic patient records, hospital, real-world data, healthcare innovation, complexity, frailty, delirium, dementia, multimorbidity, older patients, medical decision making

For peer review only - http://bmjopen.bmj.com/site/about/guidelines.xhtml

Strengths and limitations

- ORCHARD-EPR overcomes the limitations of prospective cohorts which are subject to resource constraints, recruitment challenges and selection bias (e.g. from the requirement for research consent) through providing multimodal rich clinical data at scale.
- ORCHARD-EPR has complete coverage of the target population without selection and electronic follow-up including through linkage to mental health records and brain imaging (CT, MRI, other) not routinely available in existing administrative datasets.
- ORCHARD-EPR exploits the successful regional implementation of routine on-admission cognitive screening which is often poorly captured adding further value to the rich phenotypic data collected as part of routine clinical care.
- ORCHARD-EPR is a two-centre UK study and may not be generalizable to other populations but the combined Oxfordshire and Berkshire populations are representative of England as a whole.
- Free text entries including the medical clerking, allied health assessments are currently unavailable for analysis, although these could eventually be extracted using AI tools.

For peer review only

INTRODUCTION

The advent of hospital electronic patient records (EPRs) offers the opportunity to exploit large scale routinely acquired clinical data to improve patient care. Such data offer several advantages. First, they can be obtained at scale without additional burden to patients or staff and at relatively low cost. Second, the use of EPR data (with the appropriate governance and ethical approvals) allows the entire hospital population to be studied without the unavoidable selection bias incurred in requiring individual consent for research assessments [1]. This is particularly advantageous in studies on older, frail and cognitively impaired patients in whom capacity to consent to research may be lacking, or ability to tolerate research assessments may be limited [2,3]. Third, obtaining data from the EPRs provides a considerably richer source of data than is currently available from hospital administrative datasets that are comprised largely of ICD-10 coded diagnoses which have limitations especially in capturing complexity including frailty syndromes [2,4,5]. Fourth, EPRs provide real time information allowing implementation of algorithms to identify those at high risk (e.g. of delirium) [6].

The case-mix of general hospitals is changing across high income countries in line with population ageing with implications for a wide range of hospital specialties [7-9]. Older people aged $\geq$65 years occupy the majority of hospital bed-days and many have complex conditions [10]. Up to a half have a cognitive disorder (e.g. delirium, dementia) and around 40% are physically frail with an increased vulnerability to stressors such as a sudden decline in health [2,11-16]. Both cognitive and physical frailty are associated with a broad range of adverse health and social outcomes [2,11,13,15,16] and improving acute hospital care for older patients is a priority as reflected in policy documents and guidelines internationally [17-22]. Therefore, hospital data systems need to be established to identify frail patients across the hospital and across specialties, discriminate cognitive from physical frailty and ideally, the frailty domains affected given the implications for care[2,11,17-22]. In addition, collection of detailed clinical data including on diagnosis, comorbidities and illness acuity will aid understanding of the prognostic value of frailty across a range of acute hospital specialties and settings, improve risk stratification, inform secondary and tertiary prevention measures and provide data for research [2,11,20].

In this protocol, we describe the methods underpinning the Oxford and Reading Comorbidity Frailty and Ageing Research Database - Electronic Patient Records (ORCHARD-EPR) which uses routinely acquired rich multimodal electronic individual patient data including routine

For peer review only

cognitive/delirium screening, nursing risk assessments (falls risk, nutrition, pressure sore risk), observations, laboratory tests and brain imaging. ORCHARD-EPR will facilitate healthcare innovation and medical decision making including the development/validation of risk prediction algorithms including for delirium and future dementia. ORCHARD-EPR will also enable key evidence gaps to be addressed around cognitive and physical frailty in the hospital setting as summarised in **Table 1** overcoming the limitations in existing studies from small selected samples (prospective studies) or reliance on hospital diagnostic ICD-10 coding (large retrospective studies) [4,5].

Aims and objectives

The primary aim of ORCHARD-EPR is to use individual patient EPRs to study the prevalence, complications and outcomes of cognitive and physical frailty in a large unselected, consecutive cohort of adults with unplanned hospital admission or emergency ambulatory care assessment.

The main objectives are to:

1. Estimate the prevalence of cognitive frailty (i.e. delirium, dementia, delirium superimposed on dementia, low cognitive test score) by age, sex, and specialty.
2. Estimate the prevalence of physical/global frailty using a variety of measures, including global scores (Clinical Frailty Scale [23]), and administrative diagnostic coding (ICD-10) data (Hospital Frailty Risk Score (HFRS) [16], coded frailty syndromes [24]) by age, sex, and specialty.
3. Determine the associations of cognitive and physical frailty with outcomes (death, length of stay, readmission, increased care needs at discharge) adjusted for demographics, deprivation, multimorbidity, illness severity and other putative effect modifiers [2,11].
4. Develop and integrate within the EPR a pragmatic tool (EPR-CGA) to identify frailty domains using routinely acquired clinical EPR hospital data to target frailty interventions in real-time.
5. Provide a rich research resource for further studies, including disease-specific studies (eg on stroke), time trends in frailty prevalence and outcomes, investigation of risk factors for cognitive frailty (e.g. neuroimaging markers and vascular disease), the development and validation of diagnostic and risk prediction tools (e.g. for delirium and for dementia on follow-up) and health economics studies.

For peer review only

For peer review only - http://bmjopen.bmj.com/site/about/guidelines.xhtml

METHODS AND ANALYSIS

Study design and setting

The ORCHARD-EPR database was established by collecting pseudo-anonymized routinely acquired data extracted from the Oxford University Hospitals NHS Foundation Trust (OUHFT) Cerner Millenium EPR (2015 onwards). Adult patients with unplanned admission or same day emergency care (SDEC) are included. The OUHFT is a large teaching and research trust consisting of four hospitals (John Radcliffe Hospital, Nuffield Orthopaedic Centre and Churchill Hospital in Oxford, and the Horton General Hospital in Banbury) providing all acute hospital care in Oxfordshire, UK with a catchment area of >800,000 people. There are short-stay nursing home beds for those requiring further assessment prior to discharge back to the community, and some specialty services to neighbouring counties [25]. The Oxfordshire population is older than the England average, all levels of deprivation are represented but with a lower average deprivation, and the urban/rural mix is in line with England as a whole [26]. ORCHARD-EPR is approved by the South Central Oxford C Research Ethics Committee (REC reference: 23/SC/0258). From 2023 onwards, we plan to extend ORCHARD-EPR to include the Royal Berkshire NHS Foundation Trust, with a larger catchment population (~1 million), subject to the necessary agreements. The combined Oxfordshire and Berkshire population is well-matched to the overall English population demographic characteristics.

Regulatory approvals

Extensive work (2017-current) to meet regulatory requirements and overcome logistical barriers was required (by STP) to set up ORCHARD-EPR. This has included:

- Initial OUHFT audit approval and registration to obtain the EPR data from the OUHFT Information team;
- Assigning of a unique study number (ORCHARD-EPR studyID) generated using an algorithm by the Information team (AJ) to enable de-identification of ORCHARD-EPR data;
- Extensive iterative checking of ORCHARD-EPR data accuracy in collaboration with the OUHFT Information team (AJ);
- Working with the OUHFT clinical coding team to improve ICD-10 coding for cognitive frailty including delirium [4];
- Data Protection Impact Assessment and Caldicott guardian sign-off for use of OUHFT EPR and brain imaging data and transfer of de-identified data to the University of Oxford servers;

For peer review only - http://bmjopen.bmj.com/site/about/guidelines.xhtml

- Ethics approval to use ORCHARD-EPR for research purposes with opt-out publicised on the OUHFT website and via posters;
- Approval from the Clinical Records Interactive Search (CRIS) oversight group for use of Oxford Health NHS Foundation Trust CRIS, Powered by Akrivia Health, mental health data in linkage to ORCHARD-EPR OUHFT data [27];
- Data transfer agreement between Oxford Health NHS Foundation Trust and OUHFT for transfer of pseudonymised mental health CRIS data;
- Collaboration agreement between University of Oxford and OUHFT to cover use of OUHFT data in research that might generate IP;
- Development of a pipeline to automatically de-identify, link to study ID and transfer brain scan images from the OUHFT Picture Archiving and Communication System (PACS) to the University of Oxford servers.

Participants and eligibility

The EPR was introduced to OUHFT in 2015 but complete coverage of all clinical areas and for all functionalities was not achieved until 2017. ORCHARD-EPR contains data on eligible patients from January 2015 with data added every six months and includes data covering the period of the Covid-19 pandemic and beyond.

Inclusion: OUHFT in-patients and acute ambulatory care (SDEC) patients ≥65 years, as well as patients <65 years with a completed cognitive screen or test or who are at risk of cognitive or physical frailty (e.g. patients seen in OUHFT memory/cognitive disorder clinics, vascular specialty services or with multi-morbidity). Individual consent is not obtained for inclusion of pseudonymised data in the database although patients may opt out as stated earlier.

Exclusion: Adult patients <65 years without a completed cognitive screen or test or those not otherwise at-risk of cognitive or physical frailty.

Study size

Over 200,000 unplanned hospital admissions episodes and ambulatory care assessments are included in ORCHARD-EPR (2015-to date, with data collection ongoing). Of these, 129,248 are inpatient admissions with a LOS ≥1 day in over 64,641 unique patients.

Study procedures

All ORCHARD-EPR data are obtained from assessments completed by medical, nursing and allied health professional or administrative staff as part of routine patient care. Data are

For peer review only - http://bmjopen.bmj.com/site/about/guidelines.xhtml

obtained from the EPR via the OUHFT information team or manually (>100 data fields in total, **Figure 1**). Only information available in categorical or numeric form including EPR powerforms is obtained – free text entries (e.g. powernote entries by clinicians) are not currently available. Individual patient records are assigned an ORCHARD-EPR study number by the Information Team and patient identifiers (e.g. name, date of birth, hospital MRN, NHS number, etc.) are removed before transfer to the database.

Table 2 summarises the data items collected in ORCHARD-EPR and whether they are available at multiple time-points over an admission, and how these compare with the data available from UK-wide Hospital-Episode Statistics-HES which are similar to most international hospital administrative datasets. Key datafields extracted included demographics, postcodes, treating specialty, diagnoses, cognition, nutrition, falls and pressure sore risk, observations, illness severity, laboratory analyses and outcomes including length of stay. Importantly, ORCHARD-EPR contains extensive cognitive phenotyping data obtained through embedded hospital routine cognitive screening and linkage to mental health data as well as brain imaging.

Cognitive screen

ORCHARD-EPR exploits previous extensive work (2010-) led by STP to develop, validate and implement routine cognitive screening including for delirium for at-risk OUHFT patients (**Figure 2** and see below) [4,5,15,28-33] Since 2013, OUHFT has mandated that all patients with unplanned admission ≥70 years of age or younger patients at high-risk (e.g. alcohol excess, multiple sclerosis, stroke history) are administered the validated cognitive screen on admission [4,15]. This was initially delivered as part of a paper clerking proforma (2013-2015) designed by STP and SSi but since 2015, this has been integrated as a power form into the EPR (28,466/40,439 (70%) eligible patients staying >1 day screened for period 2017-2019) [34]. Ongoing performance feedback and governance reviews are in place to ensure that screening rates are maintained.

The OUHFT cognitive screening EPR powerform (**Supplementary Figure 1**) includes:

- The 10-point Abbreviated Mental Test Score (AMTS [35]), a brief screen for cognitive problems or drop down list for reason for lack of completion (e.g. patient too unwell, aphasia);
- Recording of formal dementia diagnosis (recorded as yes/no/uncertain);
- Recording of delirium diagnosis (recorded as yes/no/uncertain) informed by the CAM [36]. Since screening is mandated on admission, prevalent (on-admission) delirium is more likely to be recorded via the powerform than incident delirium (arising during admission) [4]

For peer review only - http://bmjopen.bmj.com/site/about/guidelines.xhtml

Incident delirium may be recorded via launching another cognitive screening powerform or may be documented in free text powernote documentation which is then assigned an ICD-10 delirium code by coding staff (**Figure 1**).

Training in delirium diagnosis, using the CAM, and according to the Diagnostic and Statistical Manual of Mental Disorders, (DSM-IV, now DSM-5) criteria, is provided to junior medical staff during foundation and middle grade training and this together with Trust-wide delirium education and awareness raising, was implemented as part of a multicomponent intervention [4]: staff education/training including of the coding team, implementation of mandatory cognitive screening via the clerking proforma and then the EPR powerform, and introduction of regular performance feedback on rates of screening at hospital governance meetings, **Figure 2**. Other cognitive screening tests are built as powerforms in EPR including the mini-mental-state examination (MMSE [37]), Montreal Cognitive Assessment (MoCA [38]), Addenbrooke's Cognitive Examination (ACE-III [39]), and Informant Questionnaire for Cognitive Decline in the Elderly (IQCODE [40]) are these also included in ORCHARD-EPR where available.

Nursing Risk Assessments

ORCHARD-EPR includes data from relevant nursing risk assessments, including the Malnutrition Universal Screening Tool (MUST [41]), Braden Scale for pressure sore risk[42], and a multifactorial falls risk assessment based on National Institute for Health and Care Excellence (NICE) guidance [43]. These include data on functional status, vision and continence.

Derived variables

Laboratory test results and physiologic measures, including heart rate, blood pressure and temperature, from routine patient observations on admission are included allowing for the calculation of derived variables. These data are used to calculate measures of illness severity, including the:

- National Early Warning Score (NEWS) [44];
- Systemic Inflammatory Response Syndrome (SIRS) criteria [45] (white cell count, blood pressure , respiratory rate, temperature);
- Acute Physiology and Chronic Health Evaluation II (APACHE II) [46,47];
- Quick Sequential Organ Failure Assessment (qSOFA) score [48];

Since such data are available throughout admission, they can be used to generate dynamic data on intra-admission trajectories. ICD-10 codes are allocated by OUHFT coding

For peer review only - http://bmjopen.bmj.com/site/about/guidelines.xhtml

administrators and are used to calculate the Charlson co-morbidity score with updated weights[49],and the HFRS [16] Post codes are collected as part of admission demographics and allow calculation of the English Index of Multiple Deprivation (2019) to obtain percentiles of deprivation [50]. Post codes are also used to cross-check against social services lists of care homes to assign care home residence pre-admission [51].

Data linkage

ORCHARD-EPR linkage to the Oxford Vascular Study (OXVASC) TIA/stroke cohorts has already provided long-term follow-up data on hospital admission after TIA and stroke to inform delirium and future dementia risk associations [29,30].

ORCHARD-EPR will also be linked to:

- local mental health trust data (Oxford-Health-CRIS);
- the OUHFT incident reporting database (Ulysses);
- Criteria for Screening and Triaging to Appropriate aLternative care (CRiSTAL) scores collected by the OUHFT Palliative care team [52];
- routinely acquired CT and MRI brain imaging.

Oxford-Health CRIS contains de-identified, pseudonymised data on mental health diagnoses including dementia, mild cognitive impairment, cognitive test scores, depression and other psychiatric diagnoses including extracted from free-text inpatient and outpatient clinical records using artificial intelligence methods [27]. Linkage to CRIS will allow reliable identification of dementia pre-admission and on follow-up. Brain scan images (n>1300 linked CT- and MRI-brain scans to date) will allow incorporation of brain ageing/neuropathology markers. Linkage to the OUHFT incident reporting database (Ulysses) will provide data on adverse events in hospital, including falls (severity, associated harm), pressure ulcers and violent patient behaviours. Linkage of ORCHARD-EPR to other OUHFT and University of Oxford-approved studies will (if approvals obtained) provide data on acute hospital illness and cognitive and physical frailty, where participants have given consent for access to their medical records.

Outcome measures

- Data are collected for the following outcomes [2,20]:mortality;
- length of stay;
- delayed transfers in care (DTOC);
- discharge destination;

For peer review only - http://bmjopen.bmj.com/site/about/guidelines.xhtml

- readmissions;
- cognitive decline and dementia (from ORCHARD-EPR readmissions, and Oxford Health CRIS).

Mortality data are obtained from the Office of National Statistics, which registers all deaths in the UK. Mortality in-hospital, and at 30-, 120- and 365- days after discharge and on long-term follow-up will be collected. Length of stay is calculated from the time of admission to the time of discharge or transfer. DTOC occur when a patient is determined to be ready to go home by their clinician and multidisciplinary team, but remains in a hospital bed, for example, because their care package is not ready. Discharge destination (to public or private care homes or other hospitals (e.g. for rehabilitation) is recorded. Readmissions will be identified from ORCHARD-EPR at 30-, 120- and 365- days after discharge. All participants are followed until death or the end of the specified study period, whichever occurs first.

Inclusion of previous audits

Additional pseudonymised data from OUHFT-approved audits around frailty and outcomes are transferred to ORCHARD-EPR. Such audit data include comparisons of the OUHFT cognitive screen with additional objective cognitive testing (e.g. MMSE, IQCODE [28,31]) and rates of delirium occurrence in acute general medicine (n=6 cycles to date) with delirium diagnosis made by a consultant physician according to the DSM-IV criteria[4,15]. These audit data will act as the reference standard in evaluating the accuracy of the ORCHARD-EPR routinely acquired real-world EPR data.

Data accuracy

ORCHARD-EPR accuracy is carefully verified using the following:

- Selection and identification of data fields for extraction is done by STP, a clinician working in acute and complex medicine who uses the EPR interface on a daily basis, and has led implementation of changes to EPR;
- Extracted data is checked for accuracy by STP in collaboration with the Information Team analysts (AJ e.g. for missing data, and sense-checking of extracted values);
- Extracted data is verified against prospective audit data (e.g. for rates of delirium and diagnosed dementia [15]) and against data extracted independently by the Information team for other purposes (e.g. monthly cognitive screening rates).

For peer review only

Statistical analyses

Demographic and patient characteristics will be presented using descriptive statistics (chi-squared for categorical data and t-test/ANOVA for continuous data as appropriate). Prevalence of cognitive frailty syndromes (i.e. any cognitive syndrome/deficit, dementia, delirium, delirium superimposed on dementia, low objective test score) will be calculated overall and stratified by age, sex and specialty with 95% confidence intervals. To determine the association between frailty syndromes (independent variables) and the outcomes listed above (dependent variables), risk ratios will be modelled using logistic, Poisson and Cox regression. Effect modification and confounding by age, sex, deprivation, comorbidity, residence at the time of admission and illness severity will be evaluated. Statistical analysis methods will be updated over time, as new data are added and research questions identified.

Adherence to reporting guidelines

All research will be reported in accordance with relevant guidelines including STROBE (Strengthening The Reporting of OBservational Studies in Epidemiology)[53] and TRIPOD (Transparent Reporting of a multivariable prediction model for individual prognosis or diagnosis) guidelines[54].

Patient and Public Involvement

ORCHARD-EPR has been informed since its inception by a PPI Group, set up in 2015 by STP, to help develop our studies on cognitive and physical frailty. PPI members provided input into the ORCHARD-EPR proposal and objectives. The PPI Group comprises patients with cognitive and physical frailty with lived experience of hospitalisation and their carers a retired GP, Alzheimer's society representatives and a PPI lead with experience in frailty and dementia studies. Discussions with the PPI Group highlighted that a key concern for patients and families is the need for better information about prognosis in relation to frailty and confusion in hospital. The PPI Group has also facilitated several projects that have impacted the richness and depth of ORCHARD-EPR data including the system-wide multicomponent intervention to improve the recognition, recording and diagnostic coding of delirium and other cognitive frailty syndromes, implementation of cognitive screening in acutely unwell patients and development of the delirium susceptibility score, now implemented as an EPR digital algorithm to flag patients at-risk in real time [4,6,15].

For peer review only - http://bmjopen.bmj.com/site/about/guidelines.xhtml

DISCUSSION

Summary

ORCHARD-EPR is unique in terms of its size, inclusiveness and scope (>200,000 unselected unplanned patient episodes, 2015-21) with extensive real-world multimodal routinely acquired EPR data largely unavailable in administrative datasets based on ICD-10 diagnostic coding (**Table 2)**. ORCHARD-EPR combines detailed phenotyping for cognitive and physical frailty and other clinical characteristics with physiologic and laboratory measures, illness severity, care home residence, brain scan images and outcomes including mental health diagnoses from linkage to mental health secondary care data. ORCHARD-EPR will enable large-scale studies on the prevalence and outcomes of cognitive and physical frailty including delirium, with robust adjustment for confounding thereby filling existing knowledge gaps. ORCHARD will also enable develop of risk prediction algorithms (e.g. for dementia) and disease specific studies through selection of appropriate subgroups or through linkage to existing cohorts.

Cognitive frailty

Cognitive frailty has multiple direct implications for patient care, service planning and policy including capacity to consent for procedures, medication use, discharge planning, communication and advanced directives/proactive palliative care [2,13,15,20]. Despite its importance however, reliable estimates in unselected cohorts of its prevalence (and of relevant subtypes, particularly delirium) including by specialty are scarce [2].Existing prospective studies of highly phenotyped cohorts tend to have small sample sizes and restrictive eligibility criteria resulting in exclusion of high risk groups such as those unable to give informed consent [1-3]. Retrospective administrative datasets (eg UK HES data) are large and inclusive but are usually unreliable for ascertainment of cognitive frailty because of the insensitivity of ICD-10 diagnostic coding or reliance on non-specific free text terms (eg ‘disturbed behaviour’) [2,4,5].

ORCHARD-EPR addresses the limitations of previous datasets through combining the detailed cognitive phenotyping data usually only available in small, labour intensive prospective studies with the large sample size, inclusivity and efficiency of retrospective administrative “Big Data” studies. Specifically, the value of the electronic cognitive data in ORCHARD-EPR is greatly enhanced by several factors. First, ORCHARD-EPR exploits routine hospital-wide cognitive screening including for delirium embedded via an electronic powerform, implemented using a multicomponent intervention ensuring better recognition and documentation of cognitive frailty. Second, ORCHARD-EPR ascertains cognitive frailty using both the results of the EPR cognitive

For peer review only - http://bmjopen.bmj.com/site/about/guidelines.xhtml

screen powerform and the ICD-10 diagnostic coding applied by the hospital coding team who have been trained by STP ensuring that free text powernote entries documenting cognitive frailty are translated into the appropriate ICD-10 code. Third, the accuracy of ORCHARD-EPR cognitive frailty data is assessed against reference standard data provided by prospective audit (using analogous methodology to that used in the development and validation of the HFRS)[15,16]. Fourth, linkage to secondary care mental health data provides enhanced information on dementia and other mental health conditions enhancing the sensitivity of cognitive outcomes.

Physical frailty/Frailty domains

In comparison to cognitive frailty, established methods are available to identify (global) frailty in large unselected retrospective administrative datasets. The widely used HFRS uses a combination of a broad range of administrative diagnostic (ICD-10) codes known to be present in those with frailty but is dependent on coding accuracy resulting in heterogeneity across studies [2,16]. In addition, the HFRS provides a single numeric measure of frailty (which can be stratified as mild/moderate/severe) rather than providing information on specific frailty domains and therefore cannot inform care in real time during admission. In contrast, the rich routinely acquired clinical data in ORCHARD-EPR could be used to derive a CGA [55]in which frailty is identified across a range of domains (cognition, continence, mobility, vision, nutrition, falls risk etc) without further burden to patients or staff. Such an EPR-CGA could be implemented into EPR to identify domains of need in real time to better target frailty interventions in collaboration with patients and families.

Frailty trajectories, time trends and prognosis

Reliable capture of cognitive frailty in ORCHARD-EPR will allow evaluation of the extent of overlap with physical frailty, individual frailty trajectories across multiple admissions, and time trends in frailty prevalence in which there are few studies. Hospital frailty prevalence is likely to be increasing given the ageing population, and the introduction of ambulatory care, Same Day Emergency Care-SDEC and Hospital-at-Home/virtual ward services which tend to be accessed by younger, fitter people [32]. ORCHARD-EPR will also provide a better understanding of the prognostic value of cognitive and physical frailty and their interactions with the potential for improved risk stratification [2,11]. Inclusion of more than 100 covariates allows robust adjustment for confounders and exploration of potential effect modifiers impacting outcomes such as care home residence and illness severity [2]. Linkage to other local hospital datasets

For peer review only

For peer review only - http://bmjopen.bmj.com/site/about/guidelines.xhtml

(e.g. incident reporting for in-patient falls, challenging behaviour) will provide reliable information on the impact of cognitive and physical frailty on healthcare safety and quality to inform care bundles and policy.

Data accuracy and creating a virtuous circle

Reliable data are key to NHS management, service evaluation, planning and development, audit and research and the advent of hospital EPRs enables rich routinely acquired data to be exploited. However, healthcare staff entering data will tend to prioritise immediate patient care which may impact data accuracy [56]. ORCHARD-EPR data accuracy is checked through close working between STP, a clinician on the ground using the EPR user interface on a daily basis, and the hospital Information Team (AJ). This enables selection of datafields of interest and the necessary iterative work to be undertaken to ensure datafields are correctly identified and populated. Accuracy can be further checked against the reference standard of local prospective audit data [15]. ORCHARD-EPR aims to create a virtuous circle whereby audit and research informs EPR developments, improves data quality and ultimately improves patient care (**Figure 3**). Further, since the EPR is a dynamic entity, ORCHARD-EPR can be used to drive future EPR developments to improve data capture as evidenced by the implementation of the EPR cognitive screen to improve recognition, documentation and administrative diagnostic coding of cognitive frailty. Delirium recognition has also been enhanced by the development, validation and EPR-implementation of the delirium susceptibility score algorithm to identify those at-risk in real time [6].

Additional research questions

ORCHARD-EPR will enable researchers to explore a large number of additional research questions in the acute secondary care setting in a large, representative and inclusive population of older adults. Examples include outcomes in disease-specific studies (e.g. COVID-19) and validation and recalibration of commonly used prognostic tools, (e.g. the CURB-65 tool for mortality risk in pneumonia [57]). The availability of routinely acquired unselected brain imaging will allow development of artificial intelligence (AI) tools to extract markers of brain pathology (or "brain frailty") such as cerebral small vessel disease, brain atrophy and stroke lesions in representative older patient cohorts. The resultant imaging AI tools could be integrated into clinical imaging systems at source to provide quantitative data to aid neuroradiologists/clinicians without the need for human input. Inclusion of brain imaging data with linked clinical data will enhance the development of risk prediction tools (e.g. for delirium and dementia) to improve

For peer review only - http://bmjopen.bmj.com/site/about/guidelines.xhtml

care and selection for trials and understand mechanisms. ORCHARD-EPR will facilitate development of algorithms for in-hospital falls risk prediction/prevention and an EPR-Comprehensive Geriatric Assessment (CGA) to identify frail patients and their needs in real time without the need for additional assessments to target individualised interventions [55]. Finally, ORCHARD-EPR could act as an exemplar for the Subnational Secure Data Environments currently being set-up across England to harness the power of NHS data for the benefit of patients [58].

Strengths and limitations

This paper has outlined the strengths of ORCHARD-EPR in detail. However, there are some limitations. Not all patients have cognitive screening because of service pressures, or lack of clinician engagement, and screening might be less likely in certain patient groups including the severely ill resulting in non-random missingness and bias. However, screening rates have been high (~75% of those staying >/=72 hours) as documented by NHS England (2017-2019)[35] and ICD-10 coding for cognitive frailty has been substantially improved by a multicomponent intervention [4]. Second, the Clinical Frailty Scale [23] was only introduced during the Covid pandemic (2020 onwards) and is only now becoming routinely administered. Third, patients <65 years are only included if a cognitive screen was completed or if they are at-risk of frailty/multimorbidity. Third, free text entries including the medical clerking and entries from allied health and specialist personnel are currently unavailable, although AI methods could eventually be used to extract analyzable data as is done for mental health (CRIS) data [27].

Conclusions

ORCHARD-EPR is an ongoing large hospital research database containing multimodal highly detailed individual patient data on older individuals across all specialties including SDEC. ORCHARD-EPR will allow reliable estimation of the burden and outcomes of cognitive and physical frailty necessary for service planning and development in line with national NHS requirements. ORCHARD-EPR will also allow development of EPR implementation of algorithms to help target interventions in real-time, and inform medical decision making as well as a broad variety of clinical, service and health economic studies. Ongoing close working between academic clinicians and data analysts will enable a “learning health systems approach” with the potential to improve data quality and patient care.

ETHICS AND DISSEMINATION

For peer review only - http://bmjopen.bmj.com/site/about/guidelines.xhtml

See ‘regulatory approvals’ heading above—ORCHARD-EPR is approved by the South Central Oxford C Research Ethics Committee (REC reference: 23/SC/0258). Results from this study will be disseminated through peer-reviewed publications, presentations at national and international conferences, as well as regional meetings to improve hospital data quality and clinical services.

List of abbreviations

ACE-III: Addenbrooke’s Cognitive Examination
AMTS: Abbreviated Mental Test Score
APACHE II: Acute Physiology and Chronic Health Evaluation
CAM: Confusion Assessment Method
CGA: Comprehensive Geriatric Assessment
CRIS: Clinical Records Interactive Search
CRiSTAL: Criteria for Screening and Triaging to Appropriate aLternative care
DSM: Diagnostic and Statistical Manual of Mental Disorders
DTOC: delayed transfers in care
EPR: electronic patient records
HES: Hospital-Episode Statistics
HFRS: Hospital Frailty Risk Score
IQCODE: Informant Questionnaire for Cognitive Decline in the Elderly
MUST: Malnutrition Universal Screening Tool
MMSE: mini-mental-state examination
MoCA: Montreal Cognitive Assessment
NEWS: National Early Warning Score
NICE: National Institute for Health and Care Excellence
ORCHARD-EPR: The Oxford and Reading Cognitive Comorbidity, Frailty and Ageing Research Database - Electronic Patient Records
OUHFT: Oxford University Hospitals NHS Foundation Trust
PACS: Picture Archiving and Communication System
qSOFA: Quick Sequential Organ Failure Assessment
SDEC: same day emergency care
SIRS: Systemic Inflammatory Response Syndrome
STROBE: Strengthening The Reporting of OBservational Studies in Epidemiology

For peer review only

TRIPOD: Transparent Reporting of a multivariable prediction model for individual prognosis or diagnosis

For peer review only

For peer review only - http://bmjopen.bmj.com/site/about/guidelines.xhtml

Declarations

- **Ethics approval and consent to participate**

Waiver of consent has been granted by the South Central Oxford C Research Ethics Committee (REC reference: 23/SC/0258; 2023-2028).

- **Consent for publication**

All authors have consented to publication

- **Availability of data and materials**

Data sharing is not applicable to this article as no datasets were generated or analysed during the current study.

- **Competing interests**

The authors have no competing interests to declare

- **Funding**

EB is supported by the Rhodes Trust. STP is supported by the NIHR Oxford Biomedical Research Centre. The assembly of ORCHARD-EPR and its use for development of dementia risk prediction algorithms in older hospital patients is funded as part of a 5-year NIHR Invention for Innovation Programme grant: NIHR204290, 2023-2028.

- **Authors' contributions**

EB assembled and cleaned the ORCHARD-EPR data and drafted the manuscript. AJ identified data variables within the OUHFT data warehouse and extracted and pseudonimised individual patient EPR data prior to transfer to ORCHARD-EPR. SSi provided OUHFT management support to embedding OUHFT-wide cognitive screening and ensuring compliance and in the set-up of ORCHARD-EPR. JD located, de-identified, pseudonimised with studyID and securely transferred brain images from the hospital PACS system to the University of Oxford servers. TS (Head of Research Informatics) and AP (Informatics Analyst) led the linkage to Oxford Health mental health data and secure transfer of pseudonimised mental health data using data systems from the NIHR Oxford Biomedical Research Centre, KV, KW, DW provided support with obtaining OUHFT EPR data, AM provided support with the planned Royal Berkshire Hospitals data linkage. SSh provided critical input to the manuscript. STP developed and implemented OUHFT-wide routine cognitive screening, led the multicomponent intervention to improve compliance and ICD-10 coding of delirium and other cognitive frailty, conceived and set-up ORCHARD-EPR, obtained funding, worked with AJ to ensure data accuracy and drafted the manuscript. All authors read and approved the final manuscript.

- **Acknowledgements**

For peer review only

We thank the OUHFT EPR and Information teams for their support in implementing changes to EPR in response to our work. This project was supported by the NIHR Oxford Biomedical Research Centre. We would also like to acknowledge the work and support of the Oxford Health Research Informatics Team, and the NIHR Oxford Health Biomedical Research Centre (NIHR203316) for the linkage with pseudonymised mental health data, which was supported by CRIS (Powered by *Akrivia Health*)

- **Disclaimer**

The views expressed are those of the authors and not necessarily those of the NHS, the NIHR or the Department of Health

For peer review only

References

1. Flaatten H, Guidet B, Jung C, Boumendil A, Leaver S, Szczeklik W, et al. Consent is a confounding factor in a prospective observational study of critically ill elderly patients. *PLOS ONE* 2022, 17:e0276386.

2. Boucher EL, Gan JM, Rothwell PM, Shepperd S, Pendlebury ST. Prevalence and outcomes of frailty in unplanned hospital admissions: a systematic review and meta-analysis of hospital-wide and general (internal) medicine cohorts. *EClinicalMedicine* 2023;59:101947.

3. Gan JM, Riley J, Basting R, Demeyere N, Pendlebury ST. Decision-making capacity in older medical in-patients: frequency of assessment and rates of incapacity by decision-type and underlying brain/mind impairment. *Age Ageing* 2023;52:afad171.

4. Pendlebury ST, Lovett NG, Thomson RJ, Smith SC. Impact of a system-wide multicomponent intervention on administrative diagnostic coding for delirium and other cognitive frailty syndromes: observational prospective study. *Clin Med* 2020;20(5):454-464.

5. Soong JTY, Ng SH, Tan KXQ, Kaubryte J, Hopper A. Variation in coded frailty syndromes in secondary care administrative data: an international retrospective exploratory study. *BMJ Open* 2022;12:e052735.

6. Pendlebury ST, Lovett NG, Smith SC, Wharton R, Rothwell PM. Delirium risk stratification in consecutive unselected admissions to acute medicine: validation of a susceptibility score based on factors identified externally in pooled data for use at entry to the acute care pathway. *Age Ageing* 2017;46(2):226-231.

7. Nichols E, Steinmetz JD, Vollset SE, Fukutaki K, Chalek J, Abd-Allah F, et al. Estimation of the global prevalence of dementia in 2019 and forecasted prevalence in 2050: an analysis for the Global Burden of Disease Study 2019. *Lancet Public Health* 2022;7:e105-e125.

8. O'Caoimh R, Sezgin D, O'Donovan MR, Molloy DW, Clegg A, Rockwood K, Liew A. Prevalence of frailty in 62 countries across the world: a systematic review and meta-analysis of population-level studies. *Age Ageing* 2021;50:96-104.

9. Blodgett JM, Rockwood K, Theou O. Changes in the severity and lethality of age-related health deficit accumulation in the USA between 1999 and 2018: a population-based cohort study. *Lancet Healthy Longev* 2021;2:e96-e104.

10. Cornwell JL, R.; Sonola, L.; Poteliakhof, E. Continuity of Care for Older Hospital Patients: A call for action. The King's Fund 2012;6-7.

11. Hoogendijk EO, Afilalo J, Ensrud KE, Kowal P, Onder G, Fried LP. Frailty: implications for clinical practice and public health. *Lancet* 2019;394:1365-1375.

For peer review only

For peer review only - http://bmjopen.bmj.com/site/about/guidelines.xhtml

12. Bickel H, Hendlmeier I, Heßler JB, Junge MN, Leonhardt-Achilles S, Weber J, Schäufele M. The Prevalence of Dementia and Cognitive Impairment in Hospitals. *Deutsches Aerzteblatt Int* 2018;115:733-740.

13. Sampson EL, Blanchard MR, Jones L, Tookman A, King M. Dementia in the acute hospital: prospective cohort study of prevalence and mortality. *Br J Psychiatry* 2009;195(1):61-66.

14. Timmons S, Manning E, Barrett A, Brady NM, Browne V, O'Shea E, et al. Dementia in older people admitted to hospital: a regional multi-hospital observational study of prevalence, associations and case recognition. *Age Ageing* 2015;44(6):993-999.

15. Pendlebury ST, Lovett N, Smith S, Dutta N, Bendon C, Lloyd-Lavery A, et al. Observational, longitudinal study of delirium in consecutive unselected acute medical admissions: age-specific rates and associated factors, mortality and re-admission. *BMJ Open* 2015;5(11):e007808-e007808.

16.Gilbert T, Neuburger J, Kraindler J, Keeble E, Smith P, Ariti C, et al. Development and validation of a Hospital Frailty Risk Score focusing on older people in acute care settings using electronic hospital records: an observational study. *Lancet* 2018; 391:1775-1782.

17. VMSzorg [Internet]. VMS Safety Management Program, Frail Elderly; 2013. Available from: http://www.vmszorg.nl/themas/kwetsbare-ouderen.

18. The Royal College of Physicians. Acute care toolkit 3: Acute care for older people living with frailty [online]. 2020. Available from: https://www.rcplondon.ac.uk/guidelines-policy/acute-care-toolkit-3-acute-care-older-people-living-frailty.

19. Carpenter C, Banerjee J, Conroy S. Holistic assessment of older people. In: Buurman B, Martin F, Conroy S, eds. Silver Book. British Geriatric Society 2021.

20. Hopper A. Geriatric Medicine. In: GIRFT Programme National Specialty Reports. Getting it Right First Time; 2021.

21.James Lind Alliance. Dementia Top 10 [online]. No date. Available from: https://www.jla.nihr.ac.uk/priority-setting-partnerships/dementia/top-10-priorities/.

22. Conroy SP, Bardsley M, Smith P, Neuburger J, Keeble E, Arora S, et al: Comprehensive geriatric assessment for frail older people in acute hospitals: the HoW-CGA mixed-methods study. *NIHR HSDR* 2019;4:4.

23. Rockwood K, Song X, MacKnight C, Bergman H, Hogan DB, McDowell I, Mitnitski A. A global clinical measure of fitness and frailty in elderly people. *CMAJ* 2005;30;173:489-95.

24. Soong J, Poots AJ, Scott S, Donald K, Woodcock T, Lovett D, Bell D. Quantifying the prevalence of frailty in English hospitals. *BMJ Open* 2015;5(10):e008456.

For peer review only

25. Oxford University Hospitals NHS Foundation Trust. Annual Report and Accounts for 1 April 2018 – 31 March 2019. 2019:32.

26. The Office of National Statistics. Estimates of the population for the UK, England, Wales, Scotland and Northern Ireland. 2021 [online]. Available from: https://www.ons.gov.uk/peoplepopulationandcommunity/populationandmigration/populationestimates/bulletins/annualmidyearpopulationestimates/mid2021.

27. Fernandes AC, Cloete D, Broadbent MT, Hayes RD, Chang CK, Jackson RG, et al. Development and evaluation of a de-identification procedure for a case register sourced from mental health electronic records. *BMC Med Inform Decis Mak* 2013;13:71.

28. Pendlebury ST, Klaus SP, Mather M, de Brito M, Wharton RM. Routine cognitive screening in older patients admitted to acute medicine: abbreviated mental test score (AMTS) and subjective memory complaint versus Montreal Cognitive Assessment and IQCODE. *Age Ageing* 2015;44:1000-1005.

29. Pendlebury ST, Thomson RJ, Welch SJV, Kuker W, Rothwell PM, Oxford Vascular Study. Utility of white matter disease and atrophy on routinely acquired brain imaging for prediction of long-term delirium risk: population-based cohort study. *Age Ageing* 2022;51(1).

30. Pendlebury ST, Thomson RJ, Welch SJV, Rothwell PM, Oxford Vascular Study. Cognitive Predictors of Delirium on Long-Term Follow-Up after TIA and Stroke: Population-Based Cohort Study. *Cerebrovasc Dis* 2022;51:288-295.

31. Emery A, Wells J, Klaus SP, Mather M, Pessoa A, Pendlebury ST. Underestimation of Cognitive Impairment in Older Inpatients by the Abbreviated Mental Test Score versus the Montreal Cognitive Assessment: Cross-Sectional Observational Study. *Dement Geriatr Cogn Dis Extra* 2020;10:205-215.

32. Elias TCN, Bowen J, Hassanzadeh R, Lasserson DS, Pendlebury ST. Factors associated with admission to bed-based care: observational prospective cohort study in a multidisciplinary same day emergency care unit (SDEC). *BMC Geriatr* 2021;21(1).

33. Phelps A, Kingston B, Wharton RM, Pendlebury ST. Routine screening in the general hospital: what happens after discharge to those identified as at risk of dementia? *Clin Med* 2017;17:395-400.

34. NHS England. Dementia Assessment and Referral 2018-2019 [online]. Available from https://www.england.nhs.uk/statistics/statistical-work-areas/dementia/dementia-assessment-and-referral-2018-19/

35. Hodkinson HM. Evaluation of a mental test score for assessment of mental impairment in the elderly. *Age Ageing* 1972;1(4).

For peer review only

36. Inouye S, van Dyck C, Alessi C, Balkin S, Siegal A, Horwitz R. Clarifying confusion: the confusion assessment method. A new method for detection of delirium. *Ann Intern Med* 1990;113(12).

37. Folstein MF, Folstein SE, McHugh PR: "Mini-mental state". A practical method for grading the cognitive state of patients for the clinician. *J Psychiatr Res* 1975;12:189-198.

38. Nasreddine ZS, Phillips NA, Bedirian V, Charbonneau S, Whitehead V, Collin I, et al. The Montreal Cognitive Assessment, MoCA: a brief screening tool for mild cognitive impairment. *J Am Geriatr Soc* 2005;53:695-699.

39. Hsieh S, Schubert S, Hoon C, Mioshi E, Hodges JR. Validation of the Addenbrooke's Cognitive Examination III in frontotemporal dementia and Alzheimer's disease. *Dement Geriatr Cogn Disord* 2013;36:242-50.

40. Jorm AF. A short form of the Informant Questionnaire on Cognitive Decline in the Elderly (IQCODE): development and cross-validation. *Psychol Med* 1994;24:145-153.

41. Elia M. Guidelines for Detection and Management of Malnutrition. Malnutrition Advisory Group, Standing Committee of British Association for Parenteral and Enteral Nutrition. Maidenhead: BAPEN 2000.

42. Bergstrom N, Braden B. Predicting pressure sores in those at risk. *Am J Nurs*;1989;89:35.

43. National Institute for Health and Care Excellence. Falls in older people: assessing risk and prevention CG161 [online]. London: NICE Guideline 2013. Available from: https://www.nice.org.uk/guidance/cg161/chapter/1-recommendations. [Accessed Sept 2023].

44. Physicians TRCo: National Early Warning Score (NEWS): Standardising the assessment of acute-illness severity in the NHS. London: The Royal College of Physicians; 2012.

45. American College of Chest Physicians/Society of Critical Care Medicine Consensus Conference. Definitions for sepsis and organ failure and guidelines for the use of innovative therapies in sepsis. *Crit Care Med* 1992;20:864-874.

46. Ticinesi A, Bastoni D, Lauretani F, Calamai S, Catalano ML, Catania P, et al: Application of the Sepsis-3 Consensus Criteria in a geriatric acute care unit: A prospective study. *Eur Geriatr Med* 2019;10 (Supplement 1):S19.

47.Knaus WA, Draper EA, Wagner DP, Zimmerman JE: APACHE II. a severity of disease classification system. *Crit Care Med* 1985;13:818-829.

48. Seymour CW, Liu VX, Iwashyna TJ, Brunkhorst FM, Rea TD, Scherag A, et al. Assessment of Clinical Criteria for Sepsis: For the Third International Consensus Definitions for Sepsis and Septic Shock (Sepsis-3). *JAMA* 2016;315:762-774.

For peer review only

49. Quan H, Li B, Couris CM, Fushimi K, Graham P, Hider P, et al. Updating and Validating the Charlson Comorbidity Index and Score for Risk Adjustment in Hospital Discharge Abstracts Using Data From 6 Countries. *Am J Epidemiol* 2011;173:676-682.
50. UK Ministry of Housing, Communities and Local Government. English indices of deprivation [online]. Available from: https://www.gov.uk/government/collections/english-indices-of-deprivation.
51. Care Quality Commission. Using CQC data. 2023. Available from: https://www.cqc.org.uk/about-us/transparency/using-cqc-data.
52. Cardona-Morrell M, Hillman K. Development of a tool for defining and identifying the dying patient in hospital: Criteria for Screening and Triaging to Appropriate aLternative care (CriSTAL). *BMJ Support Palliat Care*;2015;5:78-90.
53. Vandenbroucke JP. Strengthening the Reporting of Observational Studies in Epidemiology (STROBE): Explanation and Elaboration. *Ann Intern Med* 2007;147(8):W.
54. Collins GS, Reitsma JB, Altman DG, Moons K. Transparent reporting of a multivariable prediction model for individual prognosis or diagnosis (TRIPOD): the TRIPOD Statement. *BMC Med* 2015;13(1):1.
55. Ellis G, Gardner M, Tsiachristas A, Langhorne P, Burke O, Harwood RH, et al. Comprehensive geriatric assessment for older adults admitted to hospital. *Cochrane Database Syst Rev* 2017;9:CD006211.
56. Shemtob L, Beaney T, Norton J, Majeed A. How can we improve the quality of data collected in general practice? *BMJ* 2023;380:e071950.
57. Lim WS, van der Eerden MM, Laing R, Boersma WG, Karalus N, Town GI, et al. Defining community acquired pneumonia severity on presentation to hospital: an international derivation and validation study. *Thorax* 2003;58:377-82.
58. Department of Health and Social Care. Policy paper: Data saves lives: Reshaping health and social care with data [online]. 2022. Available from: https://www.gov.uk/government/publications/data-saves-lives-reshaping-health-and-social-care-with-data/data-saves-lives-reshaping-health-and-social-care-with-data

For peer review only

For peer review only

For peer review only - http://bmjopen.bmj.com/site/about/guidelines.xhtml

Figure Legends

Figure 1. ORCHARD-EPR – origin of data and creation of the database showing inclusion of clinical EPR data, observations and laboratory tests, brain imaging, secondary mental health diagnoses, and relevant audits. Additionally, shows how cognitive data are ascertained in ORCHARD-EPR using the cognitive screening results together with administrative diagnostic (ICD-10) coding.

Figure 2. Development, validation and implementation of the cognitive screen. In two audit cycles undertaken in acute general medicine (AGM) in 2010 and 2012, the CAM was piloted in conjunction with first the MMSE and then the AMTS. The AMTS was then compared with the MoCA and the IQCODE to determine its sensitivity and specificity for mild and moderate/severe cognitive impairment. The cognitive screen comprising the AMTS+CAM was subsequently implemented in AGM for all those aged >/=70 years via a paper clerking proforma, and from 2015, hospital-wide via an EPR powerform.

Figure 3. Virtuous circle - the uses of ORCHARD-EPR. ORCHARD-EPR data can drive audit and research and the findings used to inform policy, commissioning and service planning/development as well as EPR developments to improve documentation and care.

For peer review only

Table 1

Knowledge gaps to be addressed using ORCHARD-EPR

Cognitive frailty

- Prevalence of all-cause and subtypes of cognitive frailty in the acute hospital population overall and by age, sex, and speciality
- Accuracy of ICD-10 coding for cognitive diagnoses
- Outcomes of all-cause cognitive frailty and by subtype (adjusted for confounding)

Overall frailty

- Prevalence in the acute hospital population overall and by age, sex, and speciality including SDEC as measured by different frailty tools (e.g. CFS, HFRS, Dr Foster score, frailty aggregate score)
- Prevalence of frailty markers identified from nursing assessments (e.g. at-risk of falls, pressure sores, and malnutrition)
- Time trends in frailty prevalence in in-patient vs SDEC populations
- Outcomes of frailty (adjusted for comorbidity burden, illness severity, comorbid dementia, care home residence)
- Prevalence and prognosis in key subgroups, i.e., care home residents, co-morbid dementia/delirium, severe illness

Healthcare innovation

- Development of predictive algorithms for delirium, and for dementia on follow-up
- Use of routinely acquired brain imaging for the development of AI tools to quantify “brain frailty” including atrophy and small vessel disease and old stroke lesions to aid dementia subtyping and risk stratification
- Real-time tracking of frail individuals through the hospital system to inform service delivery and design
- Development of a real-time, “streamlined CGA” to identify care needs using routinely acquired EPR data

For peer review only

Table 2. Data items available in ORCHARD-EPR, including at multiple time-points during admission, with comparison of availability in UK Hospital Episode Statistics (HES) administrative data predominantly composed of ICD-10 diagnostic codes.

Category	ORCHARD-EPR Variable	Available at multiple time-points in ORCHARD-EPR	Availability in administrative datasets (eg HES)
**Demographic**	Age, gender, postcode, deprivation	-	✓
**Admission**	Presenting complaint#	-	x
	Method (i.e. GP, ED), source	-	✓
**Vitals**	HR, BP, RR, SpO2, tympanic temp, AVPU	✓	x
**Illness severity**	NEWS, SIRS	✓	x
**Cognitive screen**	AMTS, MMSE, MoCA, ACE	✓	x
	Reason for cognitive untestability	✓	x
	Delirium diagnosis	✓	Limited*
	Delirium on admission (prevalent) and arising de novo during admission (incident) #	✓	x
	Pre-admission dementia diagnosis	-	Limited*
**Multimorbidity**	Charlson, Elixhauser indices	-	✓
**Frailty**	HFRS	-	✓
	Clinician's impression of frailty#	-	x
	Dr Foster Global Frailty Score	-	✓
	Frailty Markers, including vision and hearing	-	Limited
	Long-term catheter (medical/nursing notes#)	✓	x
	Catheter insertion during admission (medical/nursing notes#)	✓	x

For peer review only - http://bmjopen.bmj.com/site/about/guidelines.xhtml

**Functional status**	Nursing risk assessments	✓	x
**Nutrition**	Malnutrition universal screening tool (MUST)	✓	x
**Pressure sore risk**	Braden scale (pressure sore risk), including sensory perception, moisture, activity, mobility, nutrition, friction & shear	✓	x
**Falls risk factors (based on NICE)**	Orthostatic hypotension, appropriate footwear, safe walking unaided/with an aid, urinary incontinence or urgency, vision problems, fall history, fear of falling, environment (hazards, lighting, call bell), confusion/agitation/cognitive impairment	✓	x
**Mental Capacity**	Documented mental capacity assessment yes/no, decision assessed#, outcome of decision#	✓	x
**Course in hospital**	Ward	✓	x
	Specialty (i.e. Treatment Function, Main Specialty)	-	✓
	Procedures, including radiologic imaging	✓	✓
	Incidents in hospital	✓	x
**Diagnoses**	ICD-10 codes	-	✓
	Clinician working diagnosis and final diagnosis#	-	x
**Disposition**	Residence#	-	x
	Care needs/care package#	-	x
	Increased care needs at discharge: new care home, rehabilitation bed, intermediate care, increased care package#	✓	x
	LOS in hospital, critical care, palliative care, rehabilitation,	-	✓

For peer review only - http://bmjopen.bmj.com/site/about/guidelines.xhtml

	Delayed transfer of care, reasons for delayed transfer	✓	x
	Discharge destination	-	✓
	Death (via National Office of Statistics)	-	✓
**Labs**	Haemoglobin, white cell, neutrophil and lymphocyte counts, albumin, C-reactive protein, creatinine, eGFR, glucose, HbA1c, lactate, pH, sodium, thyroid stimulating hormone, urea	✓	x
**Brain imaging**	Brain imaging reports, raw brain scan images	✓	x
**Mortality**	During admission and on follow-up	-	✓
**Long term cognitive/mental health outcomes**	Dementia and mild cognitive impairment from mental health services assessment	✓	Limited**
	Cognitive test scores	✓	x
	Depression and mental illness	✓	Limited**

CRIS=Clinical Record Interactive Search, CRIS uses artificial intelligence to extract data from free text. *Only available via clinical coding which is known to be insensitive **Only available for patients readmitted to hospital in whom a corresponding ICD-10 diagnosis was recorded by the coders. #Only available in manual audits.

For peer review only - http://bmjopen.bmj.com/site/about/guidelines.xhtml

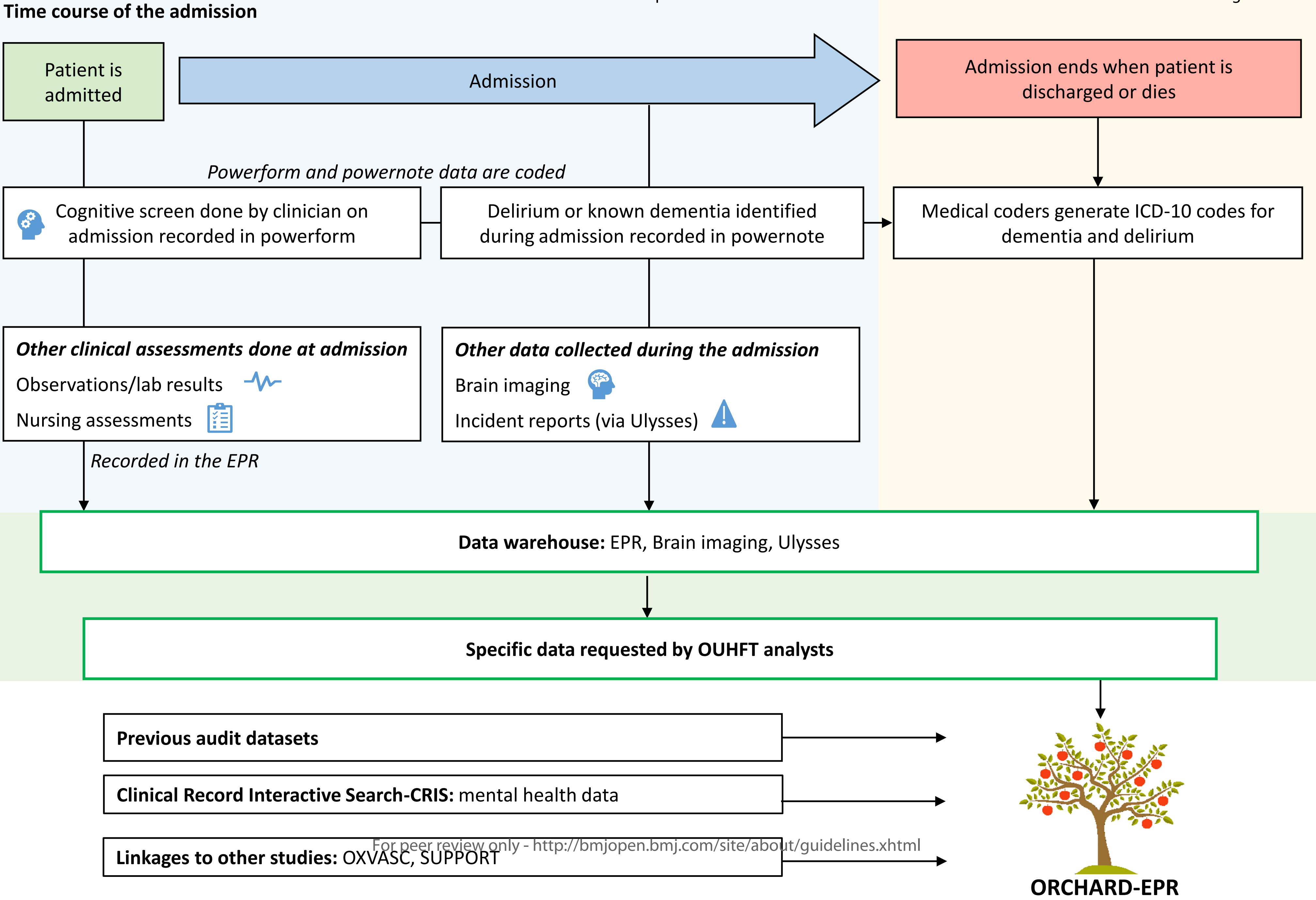

For peer review only - http://bmjopen.bmj.com/site/about/guidelines.xhtml

Development/validation/pilot of cognitive screen

- Feasibility
- MMSE vs AMTS vs MoCA vs IQCODE
- CAM for delirium

↓

Implementation, 2013

- Paper clerking proforma rolled-out for AGM
- Cognitive screen integral part of paper clerking proforma

↓

Implementation, 2015

- Roll-out of EPR
- Cognitive screen built into EPR as a powerform
- Flagged as a mandatory task on admission:
  - All patients aged ≥70 years or younger at-risk
  - Unplanned admissions
  - All specialties

↓

Performance monitoring, 2013 onwards

- Screening figures reported nationally, 2012-2019
- Feedback at clinical governance meetings
- Monthly reporting of screening compliance
- Ongoing QIP projects

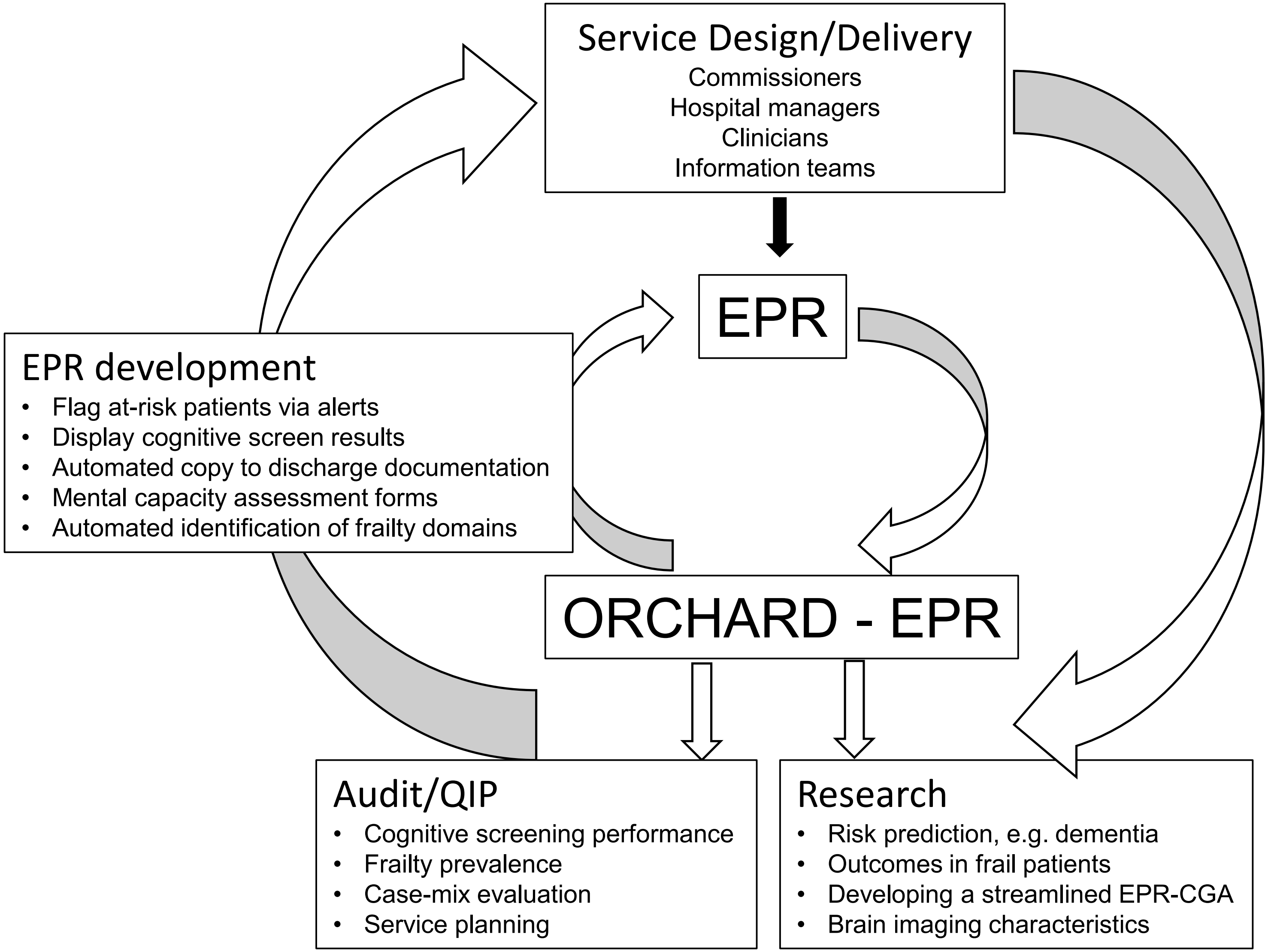
Service Design/Delivery
Commissioners
Hospital managers
Clinicians
Information teams
EPR
EPR development
• Flag at-risk patients via alerts
• Display cognitive screen results
• Automated copy to discharge documentation
• Mental capacity assessment forms
• Automated identification of frailty domains
ORCHARD - EPR
Audit/QIP
• Cognitive screening performance
• Frailty prevalence
• Case-mix evaluation
• Service planning
Research
• Risk prediction, e.g. dementia
• Outcomes in frail patients
• Developing a streamlined EPR-CGA
• Brain imaging characteristics

Supplementary material

Supplementary Figure 1 The OUHFT cognitive screening EPR powerform including the 10-point AMTS, a brief screen for cognitive problems with a drop down list for reason for lack of completion (e.g. patient too unwell, aphasia); recording of formal dementia diagnosis (recorded as yes/no/uncertain); and recording of delirium diagnosis (recorded as yes/no/uncertain) informed by the CAM.

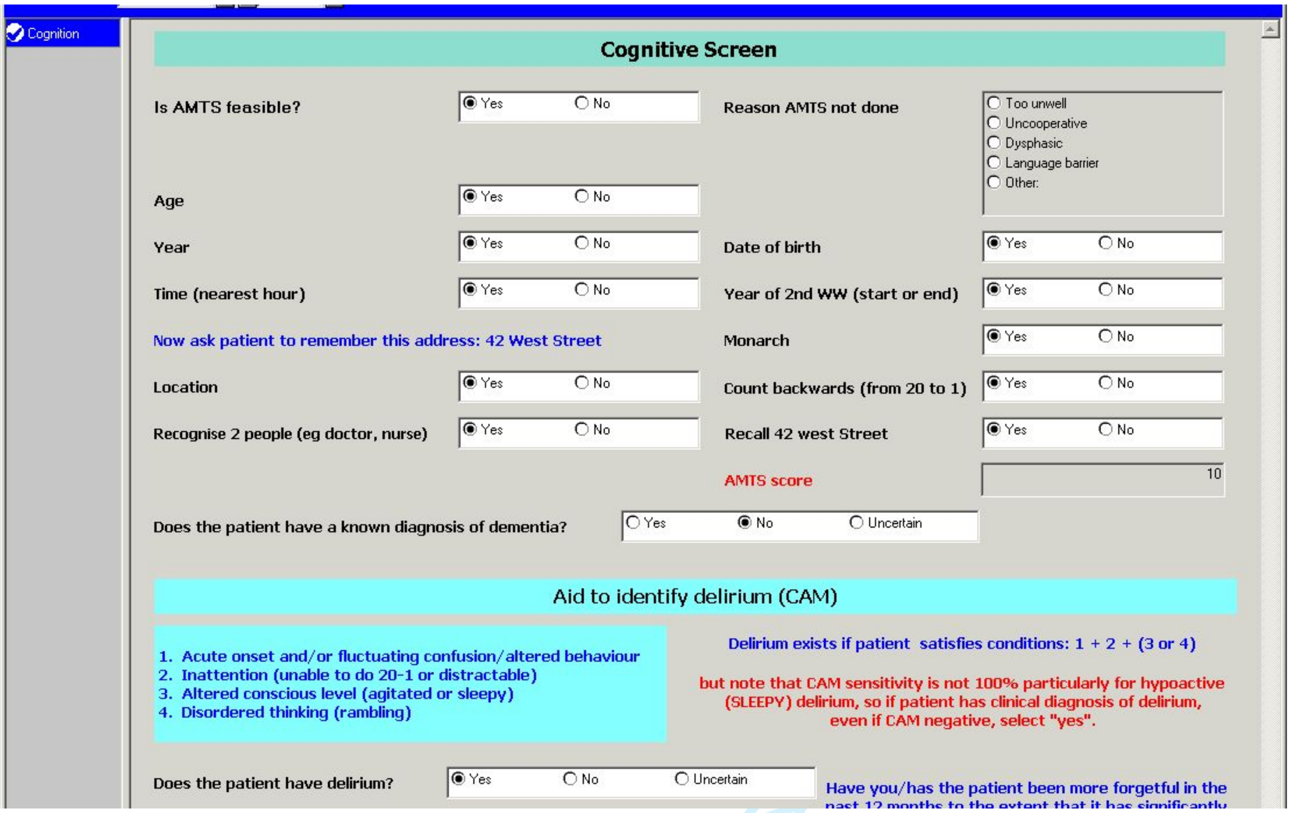

For peer review only - http://bmjopen.bmj.com/site/about/guidelines.xhtml

BMJ Open

Protocol for the Development and Analysis of the Oxford and Reading Cognitive Comorbidity, Frailty and Ageing Research Database - Electronic Patient Records (ORCHARD-EPR)

Journal:	*BMJ Open*
Manuscript ID	bmjopen-2024-085126.R1
Article Type:	Protocol
Date Submitted by the Author:	25-Apr-2024
Complete List of Authors:	Boucher, Emily; University of Oxford Wolfson Centre for Prevention of Stroke and Dementia<br>Jell, Aimee; Oxford University Hospitals NHS Foundation Trust, Informatics Department<br>Singh, Sudhir; Oxford University Hospitals NHS Foundation Trust, Departments of Acute General (Internal) Medicine and Geratology<br>Davies, Jim; University of Oxford, Department of Computer Science<br>Smith, Tanya; Oxford Health NHS Foundation Trust, Research Informatics Team, Research and Development Department; NIHR Oxford Health Biomedical Research Centre<br>Pill, Adam; Oxford Health NHS Foundation Trust, Research Informatics Team, Research and Development Department<br>Varnai, Kinga; Oxford University Hospitals NHS Foundation Trust, Research and Development Clinical Informatics<br>Woods, Kerrie; Oxford University Hospitals NHS Foundation Trust, Research and Development Clinical Informatics<br>Walliker, David; Oxford University Hospitals NHS Foundation Trust, Research and Development Clinical Informatics<br>McColl, Aubretia; Royal Berkshire NHS Foundation Trust, Departments of Acute and Elderly Care Medicine<br>Shepperd, S; University of Oxford, Nuffield Department of Population Health<br>Pendlebury, Sarah; University of Oxford Wolfson Centre for Prevention of Stroke and Dementia, Nuffield Department of Clinical Neurosciences; Oxford University Hospitals NHS Foundation Trust, Departments of Acute General (Internal) Medicine and Geratology and NIHR Oxford Biomedical Research Centre
**Primary Subject Heading**:	Geriatric medicine
Secondary Subject Heading:	Health services research, Health informatics
Keywords:	Electronic Health Records, Hospitals, Frailty, Dementia, Decision Making

Protocol for the Development and Analysis of the Oxford and Reading Cognitive Comorbidity, Frailty and Ageing Research Database - Electronic Patient Records (ORCHARD-EPR)

Emily Boucher,[a] Aimee Jell,[b] Sudhir Singh,[c] Jim Davies,[d] Tanya Smith,[e,f] Adam Pill,[e] Kinga Varnai,[g] Kerrie Woods,[g] David Walliker,[g] Aubretia McColl,[h] Sasha Shepperd,[i] Sarah T Pendlebury[a,c,j]

[a] Wolfson Centre for Prevention of Stroke and Dementia, Wolfson Building, Nuffield Department of Clinical Neurosciences, University of Oxford, UK

[b]Informatics Department, Oxford University Hospitals NHS Foundation Trust, UK

[c]Departments of Acute General (Internal) Medicine and Geratology, Oxford University Hospitals NHS Foundation Trust, UK

[d]Department of Computer Science, University of Oxford, UK

[e]Research Informatics Team, Research and Development Department, Oxford Health NHS Foundation Trust

[f]NIHR Oxford Health Biomedical Research Centre

[g]Research and Development Clinical Informatics, Oxford University Hospitals NHS Foundation Trust, UK

[h]Departments of Acute and Elderly Care Medicine, Royal Berkshire Hospitals NHS Foundation Trust

[i]Nuffield Department of Population Health, University of Oxford, UK

[j]NIHR Biomedical Research Centre, Oxford University Hospitals NHS Foundation Trust, UK

Address correspondence to: Professor Sarah Pendlebury, Wolfson Centre for Prevention of Stroke and Dementia, Wolfson Building, John Radcliffe Hospital, Oxford OX3 9DU

Email: sarah.pendlebury@ndcn.ox.ac.uk

For peer review only

Telephone: +44 1865 231603

Abstract (Max 300 words)

Background

Hospital electronic patient records (EPRs) offer the opportunity to exploit large scale routinely acquired data at relatively low cost and without selection. EPRs provide considerably richer data, and in real-time, than retrospective administrative datasets in which clinical complexity is often poorly captured. With population ageing, a wide range of hospital specialties now manage older people with multimorbidity, frailty and associated poor outcomes. We therefore set-up the Oxford and Reading Cognitive Comorbidity, Frailty and Ageing Research Database-Electronic Patient Records (ORCHARD-EPR) to facilitate clinically meaningful research in older hospital patients including algorithm development, and to aid medical decision making, implementation of guidelines, and inform policy.

Methods and analysis

ORCHARD-EPR uses routinely-acquired individual patient data on all patients aged ≥65 years with unplanned admission or Same Day Emergency Care unit attendance at four acute general hospitals serving a population of >800,000 (Oxfordshire, UK) with planned extension to the neighbouring Berkshire regional hospitals (>1,000,000). Datafields include diagnosis, comorbidities, nursing risk assessments, frailty, observations, illness acuity, laboratory tests and brain scan images. Importantly, ORCHARD-EPR contains the results from mandatory hospital-wide cognitive screening (≥70 years) comprising the 10-point Abbreviated-Mental-Test and dementia and delirium diagnosis (Confusion Assessment Method-CAM). Outcomes include length of stay, delayed transfers of care, discharge destination, readmissions, and death. The rich multimodal data are further enhanced by linkage to secondary care electronic mental health records. Selection of appropriate subgroups or linkage to existing cohorts allows disease specific studies. Over 200,000 patient episodes are included to date with data collection ongoing of which 129,248 are admissions with a LOS ≥1 day in 64,641 unique patients.

Ethics and dissemination

ORCHARD-EPR is approved by the South Central Oxford C Research Ethics Committee (ref: 23/SC/0258). Results will be widely disseminated through peer-reviewed publications and presentations at conferences, and regional meetings to improve hospital data quality and clinical services.

For peer review only - http://bmjopen.bmj.com/site/about/guidelines.xhtml

Keywords

Three to ten keywords representing the main content of the article.

Electronic patient records, hospital, real-world data, healthcare innovation, complexity, frailty, delirium, dementia, multimorbidity, older patients, medical decision making

Strengths and limitations

- ORCHARD-EPR overcomes the limitations of prospective cohorts which are subject to resource constraints, recruitment challenges and selection bias (e.g. from the requirement for research consent) through providing multimodal rich clinical data at scale.
- ORCHARD-EPR has complete coverage of the target population without selection and electronic follow-up including through linkage to mental health records and brain imaging (CT, MRI, other) not routinely available in existing administrative datasets.
- ORCHARD-EPR exploits the successful regional implementation of routine on-admission cognitive screening which is often poorly captured adding further value to the rich phenotypic data collected as part of routine clinical care.
- ORCHARD-EPR is a two-centre UK study and may not be generalizable to other populations but the combined Oxfordshire and Berkshire populations are representative of England as a whole.
- Free text entries including medical clerking and allied health assessments are currently unavailable for analysis, although these could eventually be extracted using AI tools.

For peer review only

INTRODUCTION

The advent of hospital electronic patient records (EPRs) offers the opportunity to exploit large scale routinely acquired clinical data to improve patient care. Such data offer several advantages. First, they can be obtained at scale without additional burden to patients or staff and at relatively low cost. Second, the use of EPR data (with the appropriate governance and ethical approvals) allows the entire hospital population to be studied without the unavoidable selection bias incurred in requiring individual consent for research assessments [1]. This is particularly advantageous in studies on older, frail and cognitively impaired patients in whom capacity to consent to research may be lacking, or ability to tolerate research assessments may be limited [2,3]. Third, obtaining data from the EPRs provides a considerably richer source of data than is currently available from hospital administrative datasets that are comprised largely of ICD-10 coded diagnoses which have limitations especially in capturing complexity including frailty syndromes [2,4,5]. Fourth, EPRs provide real time information allowing implementation of algorithms to identify those at high risk (e.g. of delirium) [6].

The case-mix of general hospitals is changing across high income countries in line with population ageing with implications for a wide range of hospital specialties [7-9]. Older people aged $\geq$ 65 years occupy the majority of hospital bed-days and many have complex conditions [10]. Up to a half have a cognitive disorder (e.g. delirium, dementia) and around 40% are physically frail with an increased vulnerability to stressors such as a sudden decline in health [2,11-16]. Both cognitive and physical frailty are associated with a broad range of adverse health and social outcomes [2,11,13,15,16] and improving acute hospital care for older patients is a priority as reflected in policy documents and guidelines internationally [17-22]. Therefore, hospital data systems need to be established to identify frail patients across the hospital and across specialties, discriminate cognitive from physical frailty and ideally, the frailty domains affected given the implications for care [2,11,17-22]. In addition, collection of detailed clinical data including on diagnosis, comorbidities and illness acuity will aid understanding of the prognostic value of frailty across a range of acute hospital specialties and settings, improve risk stratification, inform secondary and tertiary prevention measures and provide data for research [2,11,20].

In this protocol, we describe the methods underpinning the Oxford and Reading Comorbidity Frailty and Ageing Research Database - Electronic Patient Records (ORCHARD-EPR) which uses routinely acquired rich multimodal electronic individual patient data including routine

For peer review only - http://bmjopen.bmj.com/site/about/guidelines.xhtml

cognitive/delirium screening, nursing risk assessments (falls risk, nutrition, pressure sore risk), observations, laboratory tests and brain imaging. ORCHARD-EPR will facilitate healthcare innovation and medical decision making including the development/validation of risk prediction algorithms including for delirium and future dementia. ORCHARD-EPR will also enable key evidence gaps to be addressed around cognitive and physical frailty in the hospital setting as summarised in **Table 1** overcoming the limitations in existing studies from small selected samples (prospective studies) or reliance on hospital diagnostic ICD-10 coding (large retrospective studies) [4,5].

Aims and objectives

The primary aim of ORCHARD-EPR is to use individual patient EPRs to study the prevalence, complications and outcomes of cognitive and physical frailty in a large unselected, consecutive cohort of adults with unplanned hospital admission or emergency ambulatory care assessment.

The main objectives are to:

1. Estimate the hospital-wide prevalence of cognitive frailty (i.e. delirium, dementia, delirium superimposed on dementia, low cognitive test score) by age, sex, and specialty.
2. Estimate the prevalence of physical/global frailty using a variety of measures, including global scores (Clinical Frailty Scale [23]), and administrative diagnostic coding (ICD-10) data (Hospital Frailty Risk Score (HFRS) [16], coded frailty syndromes [24]) by age, sex, and specialty.
3. Determine the associations of cognitive and physical frailty with outcomes (death, length of stay, readmission, increased care needs at discharge) adjusted for demographics, deprivation, multimorbidity, illness severity and other putative effect modifiers [2,11].
4. Develop and integrate within the EPR a pragmatic tool (EPR-CGA) to identify frailty domains using routinely acquired clinical EPR hospital data to target frailty interventions in real-time.
5. Provide a rich research resource for further studies, including disease-specific studies (eg on stroke), time trends in frailty prevalence and outcomes, investigation of risk factors for cognitive frailty (e.g. neuroimaging markers and vascular disease), the development and validation of diagnostic and risk prediction tools (e.g. for delirium and for dementia on follow-up) and health economics studies.

For peer review only - http://bmjopen.bmj.com/site/about/guidelines.xhtml

METHODS AND ANALYSIS

Study design and setting

The ORCHARD-EPR database was established by collecting pseudo-anonymized routinely acquired data extracted from the Oxford University Hospitals NHS Foundation Trust (OUHFT) Cerner Millenium EPR (2015 onwards). Adult patients with unplanned admission or same day emergency care (SDEC) are included. The OUHFT is a large teaching and research trust consisting of four hospitals (John Radcliffe Hospital, Nuffield Orthopaedic Centre and Churchill Hospital in Oxford, and the Horton General Hospital in Banbury) providing all acute hospital care in Oxfordshire, UK with a catchment area of >800,000 people. There are short-stay nursing home beds for those requiring further assessment prior to discharge back to the community, and some specialty services to neighbouring counties [25]. The Oxfordshire population is older than the England average, all levels of deprivation are represented but with a lower average deprivation, and the urban/rural mix is in line with England as a whole [26]. ORCHARD-EPR is approved by the South Central Oxford C Research Ethics Committee (REC reference: 23/SC/0258). From 2023 onwards, we plan to extend ORCHARD-EPR to include the Royal Berkshire NHS Foundation Trust, with a larger catchment population (~1 million), subject to the necessary agreements. The combined Oxfordshire and Berkshire population is well-matched to the overall English population demographic characteristics.

Regulatory approvals

Extensive work (2017-current) to meet regulatory requirements and overcome logistical barriers was required (by STP) to set up ORCHARD-EPR. This has included:

- Initial OUHFT audit approval and registration to obtain the EPR data from the OUHFT Information team;
- Assigning of a unique study number (ORCHARD-EPR studyID) generated using an algorithm by the Information team (AJ) to enable de-identification of ORCHARD-EPR data;
- Extensive iterative checking of ORCHARD-EPR data accuracy in collaboration with the OUHFT Information team (AJ);
- Working with the OUHFT clinical coding team to improve ICD-10 coding for cognitive frailty including delirium [4];
- Data Protection Impact Assessment and Caldicott guardian sign-off for use of OUHFT EPR and brain imaging data and transfer of de-identified data to the University of Oxford servers;

For peer review only

- Ethics approval to use ORCHARD-EPR for research purposes with opt-out publicised on the OUHFT website and via posters;
- Approval from the Clinical Records Interactive Search (CRIS) oversight group for use of Oxford Health NHS Foundation Trust CRIS, Powered by Akrivia Health, mental health data in linkage to ORCHARD-EPR OUHFT data [27];
- Data transfer agreement between Oxford Health NHS Foundation Trust and OUHFT for transfer of pseudonymised mental health CRIS data;
- Collaboration agreement between University of Oxford and OUHFT to cover use of OUHFT data in research that might generate IP;
- Development of a pipeline to automatically de-identify, link to study ID and transfer brain scan images from the OUHFT Picture Archiving and Communication System (PACS) to the University of Oxford servers.
- Currently, the ethics approval for this study does not allow access to data to researchers outside the University of Oxford/OUHFT but widening access to researchers from other institutions is currently being explored.

Participants and eligibility

The EPR was introduced to OUHFT in 2015 but complete coverage of all clinical areas and for all functionalities was not achieved until 2017. ORCHARD-EPR contains data on eligible patients from January 2015 with data added every six months and includes data covering the period of the Covid-19 pandemic and beyond.

Inclusion: OUHFT in-patients and acute ambulatory care (SDEC) patients >65 years, as well as patients <65 years with a completed cognitive screen (see later), and adult out-patients with conditions increasing the risk of cognitive or physical frailty in which data have been collected as part of OUHFT-approved audits (see **Supplement**). Individual consent is not obtained for inclusion of pseudonymised data in the database although patients may opt out as stated earlier.

Exclusion: Adult patients <65 years without a completed cognitive screen or test or otherwise at risk of cognitive or physical frailty.

Study size

Over 200,000 unplanned hospital admissions episodes and ambulatory care assessments are included in ORCHARD-EPR (2015-to date, with data collection ongoing). Of these, 129,248 are inpatient admissions with a LOS ≥1 day in 64,641 unique patients.

For peer review only - http://bmjopen.bmj.com/site/about/guidelines.xhtml

Study procedures

All ORCHARD-EPR data are obtained from assessments completed by medical, nursing and allied health professional or administrative staff as part of routine patient care. Data are obtained from the EPR via the OUHFT information team or manually (>100 data fields in total, **Figure 1**). The database contains data from EPR power forms (i.e., electronic proformas from which structured data can be extracted) but not powernotes (i.e., free-text entries by clinicians, **see Supplement**). Individual patient records are assigned an ORCHARD-EPR study number by the Information Team and patient identifiers (e.g. name, date of birth, hospital MRN, NHS number, etc.) are removed before transfer to the database.

Table 2 summarises the data items collected in ORCHARD-EPR and whether they are available at multiple time-points over an admission, and how these compare with the data available from UK-wide Hospital Episode Statistics-HES which are similar to most international hospital administrative datasets. Key datafields extracted included demographics, postcodes, treating specialty, diagnoses, cognition, nutrition, falls and pressure sore risk, observations, illness severity, laboratory analyses and outcomes including length of stay. Importantly, ORCHARD-EPR contains extensive cognitive phenotyping data obtained through embedded hospital routine cognitive screening and linkage to mental health data as well as brain imaging.

Cognitive screen

ORCHARD-EPR exploits previous extensive work (2010-) led by STP to develop, validate and implement routine cognitive screening including for delirium for at-risk OUHFT patients (**Figure 2** and see below) [4,5,15,28-33]. Since 2013, OUHFT has mandated that all patients with unplanned admission ≥70 years of age or younger patients at high-risk (e.g. alcohol excess, multiple sclerosis, stroke history) are administered the validated cognitive screen on admission [4,15]. This was initially delivered as part of a paper clerking proforma (2013-2015) designed by STP and SSi but since 2015, this has been integrated as a power form into the EPR (28,466/40,439 (70%) eligible patients staying >1 day screened for period 2017-2019) [34]. Ongoing performance feedback and governance reviews are in place to ensure that screening rates are maintained.

The OUHFT cognitive screening EPR powerform (**Supplementary Figure 1**) includes:

- The 10-point Abbreviated Mental Test Score (AMTS [35]), a brief screen for cognitive problems or drop-down list for reason for lack of completion (e.g. patient too unwell, aphasia);

For peer review only - http://bmjopen.bmj.com/site/about/guidelines.xhtml

- Recording of formal dementia diagnosis (recorded as yes/no/uncertain);
- Recording of delirium diagnosis (recorded as yes/no/uncertain) informed by the CAM [36]. Since screening is mandated on admission, prevalent (on-admission) delirium is more likely to be recorded via the powerform than incident delirium (arising during admission) [4] Incident delirium may be recorded via launching another cognitive screening powerform or may be documented in free text powernote documentation which is then assigned an ICD-10 delirium code by coding staff (**Figure 1**).

Training in delirium diagnosis, using the CAM, and according to the Diagnostic and Statistical Manual of Mental Disorders, (DSM-IV, now DSM-5) criteria, is provided to junior medical staff during foundation and middle grade training and this together with Trust-wide delirium education and awareness raising, was implemented as part of a multicomponent intervention [4]: staff education/training including of the coding team, implementation of mandatory cognitive screening via the clerking proforma and then the EPR powerform, and introduction of regular performance feedback on rates of screening at hospital governance meetings, **Figure 2**. Other cognitive screening tests are built as powerforms in EPR including the mini-mental-state examination (MMSE [37]), Montreal Cognitive Assessment (MoCA [38]), Addenbrooke's Cognitive Examination (ACE-III [39]), and Informant Questionnaire for Cognitive Decline in the Elderly (IQCODE [40]) are these also included in ORCHARD-EPR where available.

Nursing Risk Assessments

ORCHARD-EPR includes data from relevant nursing risk assessments, including the Malnutrition Universal Screening Tool (MUST [41]), Braden Scale for pressure sore risk[42], and a multifactorial falls risk assessment based on National Institute for Health and Care Excellence (NICE) guidance [43]. These include data on functional status, vision and continence.

Derived variables

Laboratory test results and physiologic measures, including heart rate, blood pressure and temperature, from routine patient observations on admission are included allowing for the calculation of derived variables. These data are used to calculate measures of illness severity, including the:

- National Early Warning Score (NEWS) [44];
- Systemic Inflammatory Response Syndrome (SIRS) criteria [45] (white cell count, blood pressure, respiratory rate, temperature);

For peer review only - http://bmjopen.bmj.com/site/about/guidelines.xhtml

- Acute Physiology and Chronic Health Evaluation II (APACHE II) [46,47];
- Quick Sequential Organ Failure Assessment (qSOFA) score [48];

Since such data are available throughout admission, they can be used to generate dynamic data on intra-admission trajectories. ICD-10 codes are allocated by OUHFT coding administrators and are used to calculate the Charlson co-morbidity score with updated weights[49],and the HFRS [16] Post codes are collected as part of admission demographics and allow calculation of the English Index of Multiple Deprivation (2019) to obtain percentiles of deprivation [50]. Post codes are also used to cross-check against social services lists of care homes to assign care home residence pre-admission [51].

Data linkage

ORCHARD-EPR linkage to the Oxford Vascular Study (OXVASC) TIA/stroke cohorts has already provided long-term follow-up data on hospital admission after TIA and stroke to inform delirium and future dementia risk associations [29,30].

ORCHARD-EPR will also be linked to:

- local mental health trust data (Oxford-Health-CRIS);
- the OUHFT incident reporting database (Ulysses);
- Criteria for Screening and Triaging to Appropriate aLternative care (CRiSTAL) scores collected by the OUHFT Palliative care team [52];
- routinely acquired CT and MRI brain imaging.

Oxford-Health CRIS contains de-identified, pseudonymised data on mental health diagnoses including dementia, mild cognitive impairment, cognitive test scores, depression and other psychiatric diagnoses including extracted from free-text inpatient and outpatient clinical records using artificial intelligence methods [27]. Linkage to CRIS will allow reliable identification of dementia pre-admission and on follow-up. Brain scan images (n>1300 linked CT- and MRI-brain scans to date) will allow incorporation of brain ageing/neuropathology markers. Linkage to the OUHFT incident reporting database (Ulysses) will provide data on adverse events in hospital, including falls (severity, associated harm), pressure ulcers and violent patient behaviours. Linkage of ORCHARD-EPR to other OUHFT and University of Oxford-approved studies will (if approvals obtained) provide data on acute hospital illness and cognitive and physical frailty, where participants have given consent for access to their medical records.

Outcome measures

For peer review only

Data are collected for the following outcomes [2,20]:

- Mortality obtained from the Office of National Statistics, which registers all deaths in the UK;
- Length of stay calculated from the time of admission to the time of discharge or transfer;
- Delayed transfers in care (DTOC) where a patient is determined to be ready to go home by their clinician and multidisciplinary team, but remains in a hospital bed, for example, because their care package is not ready;
- Discharge destination to public or private care homes or other hospitals (e.g. for rehabilitation);
- Readmissions identified from ORCHARD-EPR including within 30 days of discharge;
- Cognitive decline and dementia from ORCHARD-EPR readmissions and Oxford Health (CRIS) data.

Inclusion of previous audits

Additional pseudonymised data from OUHFT-approved audits around frailty and outcomes are transferred to ORCHARD-EPR. Such audit data include comparisons of the OUHFT cognitive screen with additional objective cognitive testing (e.g. MMSE, IQCODE [28,31]) and rates of delirium occurrence in acute general medicine (n=6 cycles to date) with delirium diagnosis made by a consultant physician according to the DSM-IV criteria[4,15]. These audit data will act as the reference standard in evaluating the accuracy of the ORCHARD-EPR routinely acquired real-world EPR data.

Data accuracy

ORCHARD-EPR accuracy is carefully verified using the following:

- Selection and identification of data fields for extraction is done by STP, a clinician working in acute and complex medicine who uses the EPR interface on a daily basis, and has led implementation of changes to EPR;
- Extracted data is checked for accuracy by STP in collaboration with the Information Team analysts (AJ e.g. for missing data, and sense-checking of extracted values);
- Extracted data is verified against prospective audit data (e.g. for rates of delirium and diagnosed dementia [15]) and against data extracted independently by the Information team for other purposes (e.g. monthly cognitive screening rates).

For peer review only - http://bmjopen.bmj.com/site/about/guidelines.xhtml

Statistical analyses

Demographic and patient characteristics will be presented using descriptive statistics (chi-squared for categorical data and t-test/ANOVA for continuous data as appropriate). Prevalence of cognitive frailty syndromes (i.e. any cognitive syndrome/deficit, dementia, delirium, delirium superimposed on dementia, low objective test score) will be calculated overall and stratified by age, sex and specialty with 95% confidence intervals. To determine the association between frailty syndromes (independent variables) and the outcomes listed above (dependent variables), risk ratios will be modelled using logistic, Poisson and Cox regression. Effect modification and confounding by age, sex, deprivation, comorbidity, residence at the time of admission and illness severity will be evaluated. Statistical analysis methods will be updated over time, as new data are added and research questions identified.

Adherence to reporting guidelines

All research will be reported in accordance with relevant guidelines including STROBE (Strengthening The Reporting of OBservational Studies in Epidemiology)[53] and TRIPOD (Transparent Reporting of a multivariable prediction model for individual prognosis or diagnosis) guidelines[54].

Patient and Public Involvement

ORCHARD-EPR has been informed since its inception by a PPI Group, set up in 2015 by STP, to help develop our studies on cognitive and physical frailty. PPI members provided input into the ORCHARD-EPR proposal and objectives. The PPI Group comprises patients with cognitive and physical frailty with lived experience of hospitalisation and their carers a retired GP, Alzheimer's society representatives and a PPI lead with experience in frailty and dementia studies. Discussions with the PPI Group highlighted that a key concern for patients and families is the need for better information about prognosis in relation to frailty and confusion in hospital. The PPI Group has also facilitated several projects that have impacted the richness and depth of ORCHARD-EPR data including the system-wide multicomponent intervention to improve the recognition, recording and diagnostic coding of delirium and other cognitive frailty syndromes, implementation of cognitive screening in acutely unwell patients and development of the delirium susceptibility score, now implemented as an EPR digital algorithm to flag patients at risk in real time [4,6,15].

For peer review only - http://bmjopen.bmj.com/site/about/guidelines.xhtml

DISCUSSION

Summary

ORCHARD-EPR is unique in terms of its size, inclusiveness and scope (>200,000 unselected unplanned patient episodes, 2015-21) with extensive real-world multimodal routinely acquired EPR data largely unavailable in administrative datasets based on ICD-10 diagnostic coding (**Table 2)**. ORCHARD-EPR combines detailed phenotyping for cognitive and physical frailty and other clinical characteristics with physiologic and laboratory measures, illness severity, care home residence, brain scan images and outcomes including mental health diagnoses from linkage to mental health secondary care data. ORCHARD-EPR will enable large-scale studies on the prevalence and outcomes of cognitive and physical frailty including delirium, with robust adjustment for confounding thereby filling existing knowledge gaps. ORCHARD will also enable develop of risk prediction algorithms (e.g. for dementia) and disease specific studies through selection of appropriate subgroups or through linkage to existing cohorts.

Cognitive frailty

Cognitive frailty has multiple direct implications for patient care, service planning and policy including capacity to consent for procedures, medication use, discharge planning, communication and advanced directives/proactive palliative care [2,13,15,20]. Despite its importance however, reliable estimates in unselected cohorts of its prevalence (and of relevant subtypes, particularly delirium) including by specialty are scarce [2].Existing prospective studies of highly phenotyped cohorts tend to have small sample sizes and restrictive eligibility criteria resulting in exclusion of high risk groups such as those unable to give informed consent [1-3]. Retrospective administrative datasets (eg UK HES data) are large and inclusive but are usually unreliable for ascertainment of cognitive frailty because of the insensitivity of ICD-10 diagnostic coding or reliance on non-specific free text terms (eg 'disturbed behaviour') [2,4,5].

ORCHARD-EPR addresses the limitations of previous datasets through combining the detailed cognitive phenotyping data usually only available in small, labour intensive prospective studies with the large sample size, inclusivity and efficiency of retrospective administrative "Big Data" studies. Specifically, the value of the electronic cognitive data in ORCHARD-EPR is greatly enhanced by several factors. First, ORCHARD-EPR exploits routine hospital-wide cognitive screening including for delirium embedded via an electronic powerform, implemented using a multicomponent intervention ensuring better recognition and documentation of cognitive frailty. Second, ORCHARD-EPR ascertains cognitive frailty using both the results of the EPR cognitive

For peer review only - http://bmjopen.bmj.com/site/about/guidelines.xhtml

screen powerform and the ICD-10 diagnostic coding applied by the hospital coding team who have been trained by STP ensuring that free text powernote entries documenting cognitive frailty are translated into the appropriate ICD-10 code. Third, the accuracy of ORCHARD-EPR cognitive frailty data is assessed against reference standard data provided by prospective audit (using analogous methodology to that used in the development and validation of the HFRS)[15,16]. Fourth, linkage to secondary care mental health data provides enhanced information on dementia and other mental health conditions enhancing the sensitivity of cognitive outcomes.

Physical frailty/Frailty domains

In comparison to cognitive frailty, established methods are available to identify (global) frailty in large unselected retrospective administrative datasets. The widely used HFRS uses a combination of a broad range of administrative diagnostic (ICD-10) codes known to be present in those with frailty but is dependent on coding accuracy resulting in heterogeneity across studies [2,16]. In addition, the HFRS provides a single numeric measure of frailty (which can be stratified as mild/moderate/severe) rather than providing information on specific frailty domains and therefore cannot inform care in real time during admission. In contrast, the rich routinely acquired clinical data in ORCHARD-EPR could be used to derive a CGA [55]in which frailty is identified across a range of domains (cognition, continence, mobility, vision, nutrition, falls risk etc) without further burden to patients or staff. Such an EPR-CGA could be implemented into EPR to identify domains of need in real time to better target frailty interventions in collaboration with patients and families.

Frailty trajectories, time trends and prognosis

Reliable capture of cognitive frailty in ORCHARD-EPR will allow evaluation of the extent of overlap with physical frailty, individual frailty trajectories across multiple admissions, and time trends in frailty prevalence in which there are few studies. Hospital frailty prevalence is likely to be increasing given the ageing population, and the introduction of ambulatory care, Same Day Emergency Care-SDEC and Hospital-at-Home/virtual ward services which tend to be accessed by younger, fitter people [32]. ORCHARD-EPR will also provide a better understanding of the prognostic value of cognitive and physical frailty and their interactions with the potential for improved risk stratification [2,11]. Inclusion of more than 100 covariates allows robust adjustment for confounders and exploration of potential effect modifiers impacting outcomes such as care home residence and illness severity [2]. Linkage to other local hospital datasets

For peer review only

For peer review only - http://bmjopen.bmj.com/site/about/guidelines.xhtml

(e.g. incident reporting for in-patient falls, challenging behaviour) will provide reliable information on the impact of cognitive and physical frailty on healthcare safety and quality to inform care bundles and policy.

Data accuracy and creating a virtuous circle

Reliable data are key to NHS management, service evaluation, planning and development, audit and research and the advent of hospital EPRs enables rich routinely acquired data to be exploited. However, healthcare staff entering data will tend to prioritise immediate patient care which may impact data accuracy [56]. ORCHARD-EPR data accuracy is checked through close working between STP, a clinician on the ground using the EPR user interface on a daily basis, and the hospital Information Team (AJ). This enables selection of datafields of interest and the necessary iterative work to be undertaken to ensure datafields are correctly identified and populated. Accuracy can be further checked against the reference standard of local prospective audit data [15]. ORCHARD-EPR aims to create a virtuous circle whereby audit and research informs EPR developments, improves data quality and ultimately improves patient care (**Figure 3**). Further, since the EPR is a dynamic entity, ORCHARD-EPR can be used to drive future EPR developments to improve data capture as evidenced by the implementation of the EPR cognitive screen to improve recognition, documentation and administrative diagnostic coding of cognitive frailty. Delirium recognition has also been enhanced by the development, validation and EPR-implementation of the delirium susceptibility score algorithm to identify those at risk in real time [6].

Additional research questions

ORCHARD-EPR will enable researchers to explore a large number of additional research questions in the acute secondary care setting in a large, representative and inclusive population of older adults. Examples include outcomes in disease-specific studies (e.g. COVID-19) and validation and recalibration of commonly used prognostic tools, (e.g. the CURB-65 tool for mortality risk in pneumonia [57]). The availability of routinely acquired unselected brain imaging will allow development of artificial intelligence (AI) tools to extract markers of brain pathology (or "brain frailty") such as cerebral small vessel disease, brain atrophy and stroke lesions in representative older patient cohorts. The resultant imaging AI tools could be integrated into clinical imaging systems at source to provide quantitative data to aid neuroradiologists/clinicians without the need for human input. Inclusion of brain imaging data with linked clinical data will enhance the development of risk prediction tools (e.g. for delirium and dementia) to improve

For peer review only - http://bmjopen.bmj.com/site/about/guidelines.xhtml

care and selection for trials and understand mechanisms. ORCHARD-EPR will facilitate development of algorithms for in-hospital falls risk prediction/prevention and an EPR-Comprehensive Geriatric Assessment (CGA) to identify frail patients and their needs in real time without the need for additional assessments to target individualised interventions [55]. Finally, ORCHARD-EPR could act as an exemplar for the Subnational Secure Data Environments currently being set-up across England to harness the power of NHS data for the benefit of patients [58].

Strengths and limitations

This paper has outlined the strengths of ORCHARD-EPR in detail. However, there are some limitations. Not all patients have cognitive screening because of service pressures, or lack of clinician engagement, and screening might be less likely in certain patient groups including the severely ill resulting in non-random missingness and bias. However, screening rates have been high (~75% of those aged $\geq$75 years staying >/=72 hours) as documented by NHS England (2017-2019)[35] and ICD-10 coding for cognitive frailty has been substantially improved by a multicomponent intervention [4]. Second, the Clinical Frailty Scale [23] was only introduced during the Covid pandemic (2020 onwards) and is only now becoming routinely administered. Third, patients aged <70 years did not get routine cognitive screening and cognitive data are therefore limited to those deemed as at risk by clinicians. However, the age cut-off has now been lowered to 65 years and over (2024 onwards) so cognitive data will be routinely available on those aged 65-69 years going forwards. Fourth, medications are not currently captured in ORCHARD-EPR, but there are plans to include these data in the future. Finally, free text entries including the medical clerking and entries from allied health and specialist personnel are currently unavailable, although AI methods could eventually be used to extract analysable data as is done for mental health (CRIS) data [27].

ETHICS AND DISSEMINATION

See ‘regulatory approvals’ heading above—ORCHARD-EPR is approved by the South Central Oxford C Research Ethics Committee (REC reference: 23/SC/0258). Results from this study will be disseminated through peer-reviewed publications, presentations at national and international conferences, as well as regional meetings to improve hospital data quality and clinical services.

For peer review only - http://bmjopen.bmj.com/site/about/guidelines.xhtml

List of abbreviations

ACE-III: Addenbrooke's Cognitive Examination

AMTS: Abbreviated Mental Test Score

APACHE II: Acute Physiology and Chronic Health Evaluation

CAM: Confusion Assessment Method

CGA: Comprehensive Geriatric Assessment

CRIS: Clinical Records Interactive Search

CRiSTAL: Criteria for Screening and Triaging to Appropriate aLternative care

DSM: Diagnostic and Statistical Manual of Mental Disorders

DTOC: delayed transfers in care

EPR: electronic patient records

HES: Hospital-Episode Statistics

HFRS: Hospital Frailty Risk Score

IQCODE: Informant Questionnaire for Cognitive Decline in the Elderly

MUST: Malnutrition Universal Screening Tool

MMSE: mini-mental-state examination

MoCA: Montreal Cognitive Assessment

NEWS: National Early Warning Score

NICE: National Institute for Health and Care Excellence

ORCHARD-EPR: The Oxford and Reading Cognitive Comorbidity, Frailty and Ageing Research Database - Electronic Patient Records

OUHFT: Oxford University Hospitals NHS Foundation Trust

PACS: Picture Archiving and Communication System

qSOFA: Quick Sequential Organ Failure Assessment

SDEC: same day emergency care

SIRS: Systemic Inflammatory Response Syndrome

STROBE: Strengthening The Reporting of OBservational Studies in Epidemiology

TRIPOD: Transparent Reporting of a multivariable prediction model for individual prognosis or diagnosis

For peer review only

For peer review only - http://bmjopen.bmj.com/site/about/guidelines.xhtml

Declarations

- **Ethics approval and consent to participate**

Waiver of consent has been granted by the South Central Oxford C Research Ethics Committee (REC reference: 23/SC/0258; 2023-2028).

- **Consent for publication**

All authors have consented to publication

- **Availability of data and materials**

Data sharing is not applicable to this article as no datasets were generated or analysed during the current study.

- **Competing interests**

The authors have no competing interests to declare

- **Funding**

EB is supported by the Rhodes Trust. STP is supported by the NIHR Oxford Biomedical Research Centre. The assembly of ORCHARD-EPR and its use for development of dementia risk prediction algorithms in older hospital patients is funded as part of a 5-year NIHR Invention for Innovation Programme grant: NIHR204290, 2023-2028.

- **Authors' contributions**

EB assembled and cleaned the ORCHARD-EPR data and drafted the manuscript. AJ identified data variables within the OUHFT data warehouse and extracted and pseudonimised individual patient EPR data prior to transfer to ORCHARD-EPR. SSi provided OUHFT management support to embedding OUHFT-wide cognitive screening and ensuring compliance and in the set-up of ORCHARD-EPR. JD located, de-identified, pseudonimised with studyID and securely transferred brain images from the hospital PACS system to the University of Oxford servers. TS (Head of Research Informatics) and AP (Informatics Analyst) led the linkage to Oxford Health mental health data and secure transfer of pseudonimised mental health data using data systems from the NIHR Oxford Biomedical Research Centre, KV, KW, DW provided support with obtaining OUHFT EPR data, AM provided support with the planned Royal Berkshire Hospitals data linkage. SSh provided critical input to the manuscript. STP developed and implemented OUHFT-wide routine cognitive screening, led the multicomponent intervention to improve compliance and ICD-10 coding of delirium and other cognitive frailty, conceived and set-up ORCHARD-EPR, obtained funding, worked with AJ to ensure data accuracy and drafted the manuscript. All authors read and approved the final manuscript.

- **Acknowledgements**

For peer review only

We thank the OUHFT EPR and Information teams for their support in implementing changes to EPR in response to our work. This project was supported by the NIHR Oxford Biomedical Research Centre. We would also like to acknowledge the work and support of the Oxford Health Research Informatics Team, and the NIHR Oxford Health Biomedical Research Centre (NIHR203316) for the linkage with pseudonymised mental health data, which was supported by CRIS (Powered by *Akrivia Health*)

- **Disclaimer**

The views expressed are those of the authors and not necessarily those of the NHS, the NIHR or the Department of Health

For peer review only

References

1. Flaatten H, Guidet B, Jung C, Boumendil A, Leaver S, Szczeklik W, et al. Consent is a confounding factor in a prospective observational study of critically ill elderly patients. *PLOS ONE* 2022, 17:e0276386.

2. Boucher EL, Gan JM, Rothwell PM, Shepperd S, Pendlebury ST. Prevalence and outcomes of frailty in unplanned hospital admissions: a systematic review and meta-analysis of hospital-wide and general (internal) medicine cohorts. *EClinicalMedicine* 2023;59:101947.

3. Gan JM, Riley J, Basting R, Demeyere N, Pendlebury ST. Decision-making capacity in older medical in-patients: frequency of assessment and rates of incapacity by decision-type and underlying brain/mind impairment. *Age Ageing* 2023;52:afad171.

4. Pendlebury ST, Lovett NG, Thomson RJ, Smith SC. Impact of a system-wide multicomponent intervention on administrative diagnostic coding for delirium and other cognitive frailty syndromes: observational prospective study. *Clin Med* 2020;20(5):454-464.

5. Soong JTY, Ng SH, Tan KXQ, Kaubryte J, Hopper A. Variation in coded frailty syndromes in secondary care administrative data: an international retrospective exploratory study. *BMJ Open* 2022;12:e052735.

6. Pendlebury ST, Lovett NG, Smith SC, Wharton R, Rothwell PM. Delirium risk stratification in consecutive unselected admissions to acute medicine: validation of a susceptibility score based on factors identified externally in pooled data for use at entry to the acute care pathway. *Age Ageing* 2017;46(2):226-231.

7. Nichols E, Steinmetz JD, Vollset SE, Fukutaki K, Chalek J, Abd-Allah F, et al. Estimation of the global prevalence of dementia in 2019 and forecasted prevalence in 2050: an analysis for the Global Burden of Disease Study 2019. *Lancet Public Health* 2022;7:e105-e125.

8. O'Caoimh R, Sezgin D, O'Donovan MR, Molloy DW, Clegg A, Rockwood K, Liew A. Prevalence of frailty in 62 countries across the world: a systematic review and meta-analysis of population-level studies. *Age Ageing* 2021;50:96-104.

9. Blodgett JM, Rockwood K, Theou O. Changes in the severity and lethality of age-related health deficit accumulation in the USA between 1999 and 2018: a population-based cohort study. *Lancet Healthy Longev* 2021;2:e96-e104.

10. Cornwell JL, R.; Sonola, L.; Poteliakhof, E. Continuity of Care for Older Hospital Patients: A call for action. The King's Fund 2012;6-7.

11. Hoogendijk EO, Afilalo J, Ensrud KE, Kowal P, Onder G, Fried LP. Frailty: implications for clinical practice and public health. *Lancet* 2019;394:1365-1375.

For peer review only

12. Bickel H, Hendlmeier I, Heßler JB, Junge MN, Leonhardt-Achilles S, Weber J, Schäufele M. The Prevalence of Dementia and Cognitive Impairment in Hospitals. *Deutsches Aerzteblatt Int* 2018;115:733-740.

13. Sampson EL, Blanchard MR, Jones L, Tookman A, King M. Dementia in the acute hospital: prospective cohort study of prevalence and mortality. *Br J Psychiatry* 2009;195(1):61-66.

14. Timmons S, Manning E, Barrett A, Brady NM, Browne V, O'Shea E, et al. Dementia in older people admitted to hospital: a regional multi-hospital observational study of prevalence, associations and case recognition. *Age Ageing* 2015;44(6):993-999.

15. Pendlebury ST, Lovett N, Smith S, Dutta N, Bendon C, Lloyd-Lavery A, et al. Observational, longitudinal study of delirium in consecutive unselected acute medical admissions: age-specific rates and associated factors, mortality and re-admission. *BMJ Open* 2015;5(11):e007808-e007808.

16.Gilbert T, Neuburger J, Kraindler J, Keeble E, Smith P, Ariti C, et al. Development and validation of a Hospital Frailty Risk Score focusing on older people in acute care settings using electronic hospital records: an observational study. *Lancet* 2018; 391:1775-1782.

17. VMSzorg [Internet]. VMS Safety Management Program, Frail Elderly; 2013. Available from: http://www.vmszorg.nl/themas/kwetsbare-ouderen.

18. The Royal College of Physicians. Acute care toolkit 3: Acute care for older people living with frailty [online]. 2020. Available from: https://www.rcplondon.ac.uk/guidelines-policy/acute-care-toolkit-3-acute-care-older-people-living-frailty.

19. Carpenter C, Banerjee J, Conroy S. Holistic assessment of older people. In: Buurman B, Martin F, Conroy S, eds. Silver Book. British Geriatric Society 2021.

20. Hopper A. Geriatric Medicine. In: GIRFT Programme National Specialty Reports. Getting it Right First Time; 2021.

21.James Lind Alliance. Dementia Top 10 [online]. No date. Available from: https://www.jla.nihr.ac.uk/priority-setting-partnerships/dementia/top-10-priorities/.

22. Conroy SP, Bardsley M, Smith P, Neuburger J, Keeble E, Arora S, et al: Comprehensive geriatric assessment for frail older people in acute hospitals: the HoW-CGA mixed-methods study. *NIHR HSDR* 2019;4:4.

23. Rockwood K, Song X, MacKnight C, Bergman H, Hogan DB, McDowell I, Mitnitski A. A global clinical measure of fitness and frailty in elderly people. *CMAJ* 2005;30;173:489-95.

24. Soong J, Poots AJ, Scott S, Donald K, Woodcock T, Lovett D, Bell D. Quantifying the prevalence of frailty in English hospitals. *BMJ Open* 2015;5(10):e008456.

For peer review only

25. Oxford University Hospitals NHS Foundation Trust. Annual Report and Accounts for 1 April 2018 – 31 March 2019. 2019:32.

26. The Office of National Statistics. Estimates of the population for the UK, England, Wales, Scotland and Northern Ireland. 2021 [online]. Available from: https://www.ons.gov.uk/peoplepopulationandcommunity/populationandmigration/populationestimates/bulletins/annualmidyearpopulationestimates/mid2021.

27. Fernandes AC, Cloete D, Broadbent MT, Hayes RD, Chang CK, Jackson RG, et al. Development and evaluation of a de-identification procedure for a case register sourced from mental health electronic records. *BMC Med Inform Decis Mak* 2013;13:71.

28. Pendlebury ST, Klaus SP, Mather M, de Brito M, Wharton RM. Routine cognitive screening in older patients admitted to acute medicine: abbreviated mental test score (AMTS) and subjective memory complaint versus Montreal Cognitive Assessment and IQCODE. *Age Ageing* 2015;44:1000-1005.

29. Pendlebury ST, Thomson RJ, Welch SJV, Kuker W, Rothwell PM, Oxford Vascular Study. Utility of white matter disease and atrophy on routinely acquired brain imaging for prediction of long-term delirium risk: population-based cohort study. *Age Ageing* 2022;51(1).

30. Pendlebury ST, Thomson RJ, Welch SJV, Rothwell PM, Oxford Vascular Study. Cognitive Predictors of Delirium on Long-Term Follow-Up after TIA and Stroke: Population-Based Cohort Study. *Cerebrovasc Dis* 2022;51:288-295.

31. Emery A, Wells J, Klaus SP, Mather M, Pessoa A, Pendlebury ST. Underestimation of Cognitive Impairment in Older Inpatients by the Abbreviated Mental Test Score versus the Montreal Cognitive Assessment: Cross-Sectional Observational Study. *Dement Geriatr Cogn Dis Extra* 2020;10:205-215.

32. Elias TCN, Bowen J, Hassanzadeh R, Lasserson DS, Pendlebury ST. Factors associated with admission to bed-based care: observational prospective cohort study in a multidisciplinary same day emergency care unit (SDEC). *BMC Geriatr* 2021;21(1).

33. Phelps A, Kingston B, Wharton RM, Pendlebury ST. Routine screening in the general hospital: what happens after discharge to those identified as at risk of dementia? *Clin Med* 2017;17:395-400.

34. NHS England. Dementia Assessment and Referral 2018-2019 [online]. Available from https://www.england.nhs.uk/statistics/statistical-work-areas/dementia/dementia-assessment-and-referral-2018-19/

35. Hodkinson HM. Evaluation of a mental test score for assessment of mental impairment in the elderly. *Age Ageing* 1972;1(4).

For peer review only - http://bmjopen.bmj.com/site/about/guidelines.xhtml

36. Inouye S, van Dyck C, Alessi C, Balkin S, Siegal A, Horwitz R. Clarifying confusion: the confusion assessment method. A new method for detection of delirium. *Ann Intern Med* 1990;113(12).

37. Folstein MF, Folstein SE, McHugh PR: "Mini-mental state". A practical method for grading the cognitive state of patients for the clinician. *J Psychiatr Res* 1975;12:189-198.

38. Nasreddine ZS, Phillips NA, Bedirian V, Charbonneau S, Whitehead V, Collin I, et al. The Montreal Cognitive Assessment, MoCA: a brief screening tool for mild cognitive impairment. *J Am Geriatr Soc* 2005;53:695-699.

39. Hsieh S, Schubert S, Hoon C, Mioshi E, Hodges JR. Validation of the Addenbrooke's Cognitive Examination III in frontotemporal dementia and Alzheimer's disease. *Dement Geriatr Cogn Disord* 2013;36:242-50.

40. Jorm AF. A short form of the Informant Questionnaire on Cognitive Decline in the Elderly (IQCODE): development and cross-validation. *Psychol Med* 1994;24:145-153.

41. Elia M. Guidelines for Detection and Management of Malnutrition. Malnutrition Advisory Group, Standing Committee of British Association for Parenteral and Enteral Nutrition. Maidenhead: BAPEN 2000.

42. Bergstrom N, Braden B. Predicting pressure sores in those at risk. *Am J Nurs*;1989;89:35.

43. National Institute for Health and Care Excellence. Falls in older people: assessing risk and prevention CG161 [online]. London: NICE Guideline 2013. Available from: https://www.nice.org.uk/guidance/cg161/chapter/1-recommendations. [Accessed Sept 2023].

44. Physicians TRCo: National Early Warning Score (NEWS): Standardising the assessment of acute-illness severity in the NHS. London: The Royal College of Physicians; 2012.

45. American College of Chest Physicians/Society of Critical Care Medicine Consensus Conference. Definitions for sepsis and organ failure and guidelines for the use of innovative therapies in sepsis. *Crit Care Med* 1992;20:864-874.

46. Ticinesi A, Bastoni D, Lauretani F, Calamai S, Catalano ML, Catania P, et al: Application of the Sepsis-3 Consensus Criteria in a geriatric acute care unit: A prospective study. *Eur Geriatr Med* 2019;10 (Supplement 1):S19.

47.Knaus WA, Draper EA, Wagner DP, Zimmerman JE: APACHE II. a severity of disease classification system. *Crit Care Med* 1985;13:818-829.

48. Seymour CW, Liu VX, Iwashyna TJ, Brunkhorst FM, Rea TD, Scherag A, et al. Assessment of Clinical Criteria for Sepsis: For the Third International Consensus Definitions for Sepsis and Septic Shock (Sepsis-3). *JAMA* 2016;315:762-774.

For peer review only - http://bmjopen.bmj.com/site/about/guidelines.xhtml

49. Quan H, Li B, Couris CM, Fushimi K, Graham P, Hider P, et al. Updating and Validating the Charlson Comorbidity Index and Score for Risk Adjustment in Hospital Discharge Abstracts Using Data From 6 Countries. *Am J Epidemiol* 2011;173:676-682.
50. UK Ministry of Housing, Communities and Local Government. English indices of deprivation [online]. Available from: https://www.gov.uk/government/collections/english-indices-of-deprivation.
51. Care Quality Commission. Using CQC data. 2023. Available from: https://www.cqc.org.uk/about-us/transparency/using-cqc-data.
52. Cardona-Morrell M, Hillman K. Development of a tool for defining and identifying the dying patient in hospital: Criteria for Screening and Triaging to Appropriate aLternative care (CriSTAL). *BMJ Support Palliat Care*;2015;5:78-90.
53. Vandenbroucke JP. Strengthening the Reporting of Observational Studies in Epidemiology (STROBE): Explanation and Elaboration. *Ann Intern Med* 2007;147(8):W.
54. Collins GS, Reitsma JB, Altman DG, Moons K. Transparent reporting of a multivariable prediction model for individual prognosis or diagnosis (TRIPOD): the TRIPOD Statement. *BMC Med* 2015;13(1):1.
55. Ellis G, Gardner M, Tsiachristas A, Langhorne P, Burke O, Harwood RH, et al. Comprehensive geriatric assessment for older adults admitted to hospital. *Cochrane Database Syst Rev* 2017;9:CD006211.
56. Shemtob L, Beaney T, Norton J, Majeed A. How can we improve the quality of data collected in general practice? *BMJ* 2023;380:e071950.
57. Lim WS, van der Eerden MM, Laing R, Boersma WG, Karalus N, Town GI, et al. Defining community acquired pneumonia severity on presentation to hospital: an international derivation and validation study. *Thorax* 2003;58:377-82.
58. Department of Health and Social Care. Policy paper: Data saves lives: Reshaping health and social care with data [online]. 2022. Available from: https://www.gov.uk/government/publications/data-saves-lives-reshaping-health-and-social-care-with-data/data-saves-lives-reshaping-health-and-social-care-with-data

For peer review only

For peer review only

For peer review only - http://bmjopen.bmj.com/site/about/guidelines.xhtml

Figure Legends

Figure 1. ORCHARD-EPR – origin of data and creation of the database showing inclusion of clinical EPR data, observations and laboratory tests, brain imaging, secondary mental health diagnoses, and relevant audits. Additionally, shows how cognitive data are ascertained in ORCHARD-EPR using the cognitive screening results together with administrative diagnostic (ICD-10) coding.

Figure 2. Development, validation and implementation of the cognitive screen. In two audit cycles undertaken in acute general medicine (AGM) in 2010 and 2012, the CAM was piloted in conjunction with first the MMSE and then the AMTS. The AMTS was then compared with the MoCA and the IQCODE to determine its sensitivity and specificity for mild and moderate/severe cognitive impairment. The cognitive screen comprising the AMTS+CAM was subsequently implemented in AGM for all those aged >/=70 years via a paper clerking proforma, and from 2015, hospital-wide via an EPR powerform.

Figure 3. Virtuous circle - the uses of ORCHARD-EPR. ORCHARD-EPR data can drive audit and research and the findings used to inform policy, commissioning and service planning/development as well as EPR developments to improve documentation and care.

For peer review only

For peer review only - http://bmjopen.bmj.com/site/about/guidelines.xhtml

Table 1

Knowledge gaps to be addressed using ORCHARD-EPR

Cognitive frailty

- Prevalence of all-cause and subtypes of cognitive frailty in the acute hospital population overall and by age, sex, and speciality
- Accuracy of ICD-10 coding for cognitive diagnoses
- Outcomes of all-cause cognitive frailty and by subtype (adjusted for confounding)

Overall frailty

- Prevalence in the acute hospital population overall and by age, sex, and speciality including SDEC as measured by different frailty tools (e.g. CFS, HFRS, Dr Foster score, frailty aggregate score)
- Prevalence of frailty markers identified from nursing assessments (e.g. at risk of falls, pressure sores, and malnutrition)
- Time trends in frailty prevalence in in-patient vs SDEC populations
- Outcomes of frailty (adjusted for comorbidity burden, illness severity, comorbid dementia, care home residence)
- Prevalence and prognosis in key subgroups, i.e., care home residents, co-morbid dementia/delirium, severe illness

Healthcare innovation

- Development of predictive algorithms for delirium, and for dementia on follow-up
- Use of routinely acquired brain imaging for the development of AI tools to quantify "brain frailty" including atrophy and small vessel disease and old stroke lesions to aid dementia subtyping and risk stratification
- Real-time tracking of frail individuals through the hospital system to inform service delivery and design
- Development of a real-time, "streamlined CGA" to identify care needs using routinely acquired EPR data

For peer review only - http://bmjopen.bmj.com/site/about/guidelines.xhtml

Table 2. Data items available in ORCHARD-EPR, including at multiple time-points during admission, with comparison of availability in UK Hospital Episode Statistics (HES) administrative data predominantly composed of ICD-10 diagnostic codes.

Category	ORCHARD-EPR Variable	Available at multiple time-points in ORCHARD-EPR	Availability in administrative datasets (eg HES)
**Demographic**	Age, gender, ethnicity, postcode, deprivation	-	✓
**Admission**	Presenting complaint#	-	x
	Method (i.e. GP, ED), source	-	✓
**Vitals**	HR, BP, RR, SpO2, tympanic temp, AVPU	✓	x
**Illness severity**	NEWS, SIRS	✓	x
**Cognitive screen**	AMTS, MMSE, MoCA, ACE	✓	x
	Reason for cognitive untestability	✓	x
	Delirium diagnosis	✓	Limited*
	Delirium on admission (prevalent) and arising de novo during admission (incident) #	✓	x
	Pre-admission dementia diagnosis	-	Limited*
**Multimorbidity**	Charlson, Elixhauser indices	-	✓
**Frailty**	HFRS	-	✓
	Clinician's impression of frailty#	-	x
	Dr Foster Global Frailty Score	-	✓
	Frailty Markers, including vision and hearing	-	Limited
	Long-term catheter (medical/nursing notes#)	✓	x
	Catheter insertion during admission (medical/nursing notes#)	✓	x

For peer review only - http://bmjopen.bmj.com/site/about/guidelines.xhtml

**Functional status**	Nursing risk assessments	✓	x
**Nutrition**	Malnutrition universal screening tool (MUST)	✓	x
**Pressure sore risk**	Braden scale (pressure sore risk), including sensory perception, moisture, activity, mobility, nutrition, friction & shear	✓	x
**Falls risk factors (based on NICE)**	Orthostatic hypotension, appropriate footwear, safe walking unaided/with an aid, urinary incontinence or urgency, vision problems, fall history, fear of falling, environment (hazards, lighting, call bell), confusion/agitation/cognitive impairment	✓	x
**Mental Capacity**	Documented mental capacity assessment yes/no, decision assessed#, outcome of decision#	✓	x
**Course in hospital**	Ward	✓	x
	Specialty (i.e. Treatment Function, Main Specialty)	-	✓
	Procedures, including radiologic imaging	✓	✓
	Incidents in hospital	✓	x
**Diagnoses**	ICD-10 codes	-	✓
	Clinician working diagnosis and final diagnosis#	-	x
**Disposition**	Residence#	-	x
	Care needs/care package#	-	x
	Increased care needs at discharge: new care home, rehabilitation bed, intermediate care, increased care package#	✓	x
	LOS in hospital, critical care, palliative care, rehabilitation,	-	✓

For peer review only

	Delayed transfer of care, reasons for delayed transfer	✓	x
	Discharge destination	-	✓
	Death (via National Office of Statistics)	-	✓
**Labs**	Haemoglobin, white cell, neutrophil and lymphocyte counts, albumin, C-reactive protein, creatinine, eGFR, glucose, HbA1c, lactate, pH, sodium, thyroid stimulating hormone, urea	✓	x
**Brain imaging**	Brain imaging reports, raw brain scan images	✓	x
**Mortality**	During admission and on follow-up	-	✓
**Long term cognitive/mental health outcomes**	Dementia and mild cognitive impairment from mental health services assessment	✓	Limited**
	Cognitive test scores	✓	x
	Depression and mental illness	✓	Limited**

CRIS=Clinical Record Interactive Search, CRIS uses artificial intelligence to extract data from free text. *Only available via clinical coding which is known to be insensitive **Only available for patients readmitted to hospital in whom a corresponding ICD-10 diagnosis was recorded by the coders. #Only available in manual audits.

For peer review only

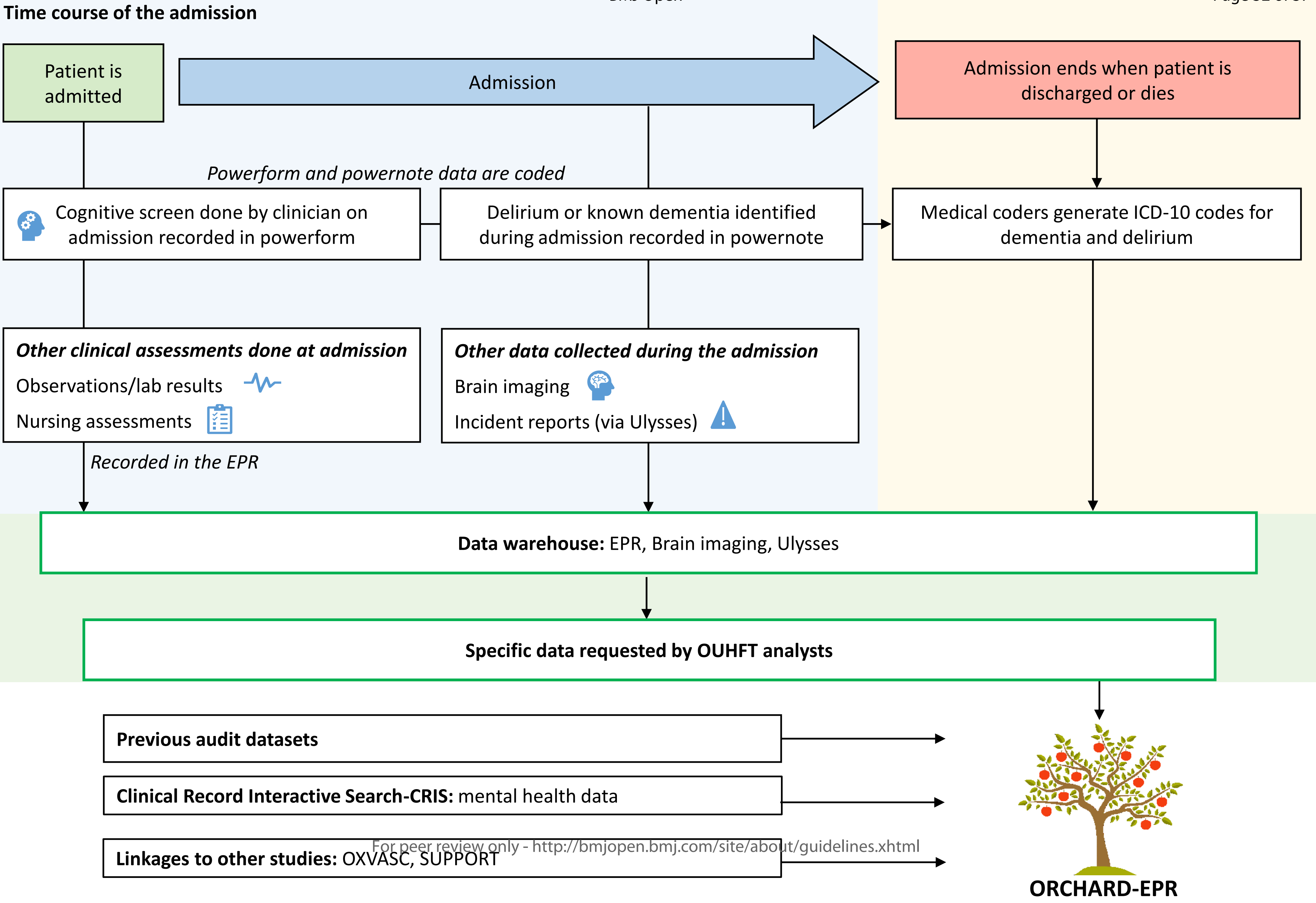

For peer review only - http://bmjopen.bmj.com/site/about/guidelines.xhtml

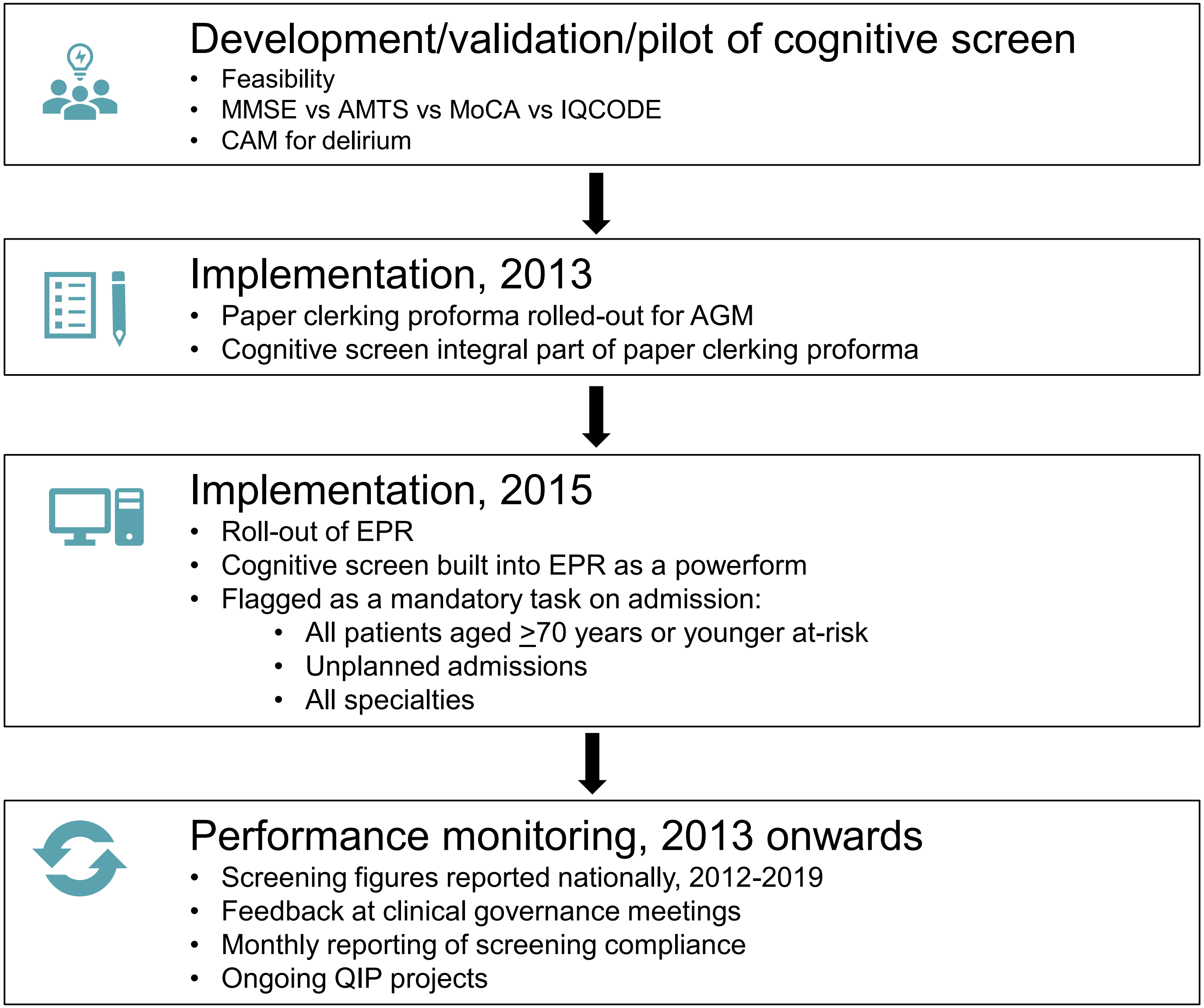

For peer review only - http://bmjopen.bmj.com/site/about/guidelines.xhtml

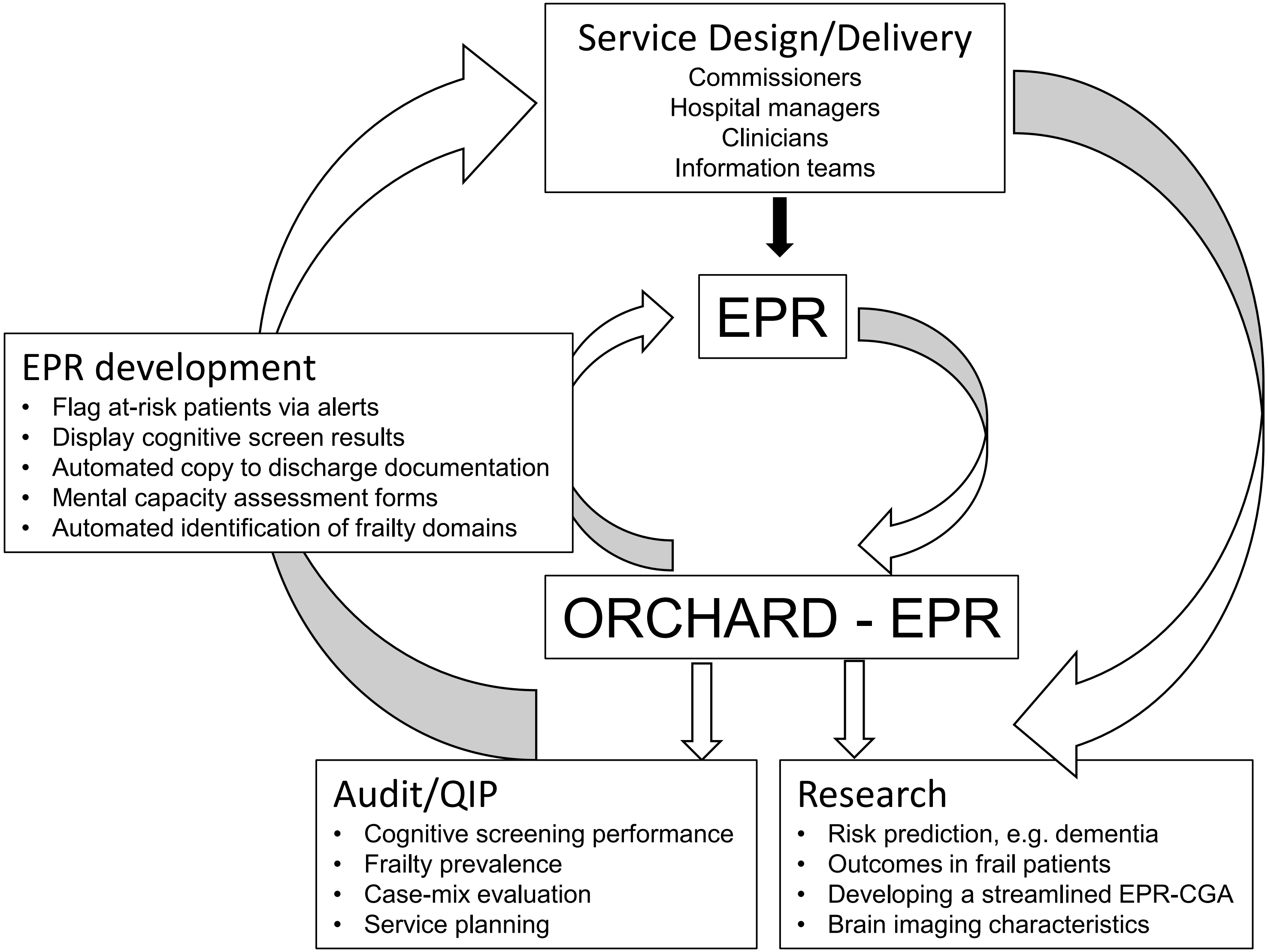
Service Design/Delivery
Commissioners
Hospital managers
Clinicians
Information teams
EPR
EPR development
• Flag at-risk patients via alerts
• Display cognitive screen results
• Automated copy to discharge documentation
• Mental capacity assessment forms
• Automated identification of frailty domains
ORCHARD - EPR
Audit/QIP
• Cognitive screening performance
• Frailty prevalence
• Case-mix evaluation
• Service planning
Research
• Risk prediction, e.g. dementia
• Outcomes in frail patients
• Developing a streamlined EPR-CGA
• Brain imaging characteristics

Protocol for the Development and Analysis of the Oxford and Reading Cognitive Comorbidity, Frailty and Ageing Research Database - Electronic Patient Records (ORCHARD-EPR).

Emily Boucher,[a] Aimee Jell,[b] Sudhir Singh,[c] Jim Davies,[d] Tanya Smith,[e,f] Adam Pill,[e] Kinga Varnai,[g] Kerrie Woods,[g] David Walliker,[g] Aubretia McColl,[h] Sasha Shepperd,[i] Sarah T Pendlebury[a,c,j]

[a] Wolfson Centre for Prevention of Stroke and Dementia, Wolfson Building, Nuffield Department of Clinical Neurosciences, University of Oxford, UK

[b]Informatics Department, Oxford University Hospitals NHS Foundation Trust, UK

[c]Departments of Acute General (Internal) Medicine and Geratology, Oxford University Hospitals NHS Foundation Trust, UK

[d]Department of Computer Science, University of Oxford, UK

[e]Research Informatics Team, Research and Development Department, Oxford Health NHS Foundation Trust

[f]NIHR Oxford Health Biomedical Research Centre

[g]Research and Development Clinical Informatics, Oxford University Hospitals NHS Foundation Trust, UK

[h]Departments of Acute and Elderly Care Medicine, Royal Berkshire Hospitals NHS Foundation Trust

[i]Nuffield Department of Population Health, University of Oxford, UK

[j]NIHR Biomedical Research Centre, Oxford University Hospitals NHS Foundation Trust, UK

Address correspondence to: Professor Sarah Pendlebury, Wolfson Centre for Prevention of Stroke and Dementia, Wolfson Building, John Radcliffe Hospital, Oxford OX3 9DU

Email: sarah.pendlebury@ndcn.ox.ac.uk

For peer review only

Supplementary material

Supplemental Methods

ORCHARD-EPR Inclusion Criteria

Patients with unplanned admission or Same Day Emergency Care-SDEC attendance

All OUHFT in-patients with unplanned admission and acute ambulatory care (Same Day Emergency Care-SDEC) patients aged ≥65 years are included in the database with the relevant datafields extracted from the EPR by the OUHFT Information analysts. Similar data are automatically extracted for in-patients and SDEC patients aged <65 years with a completed cognitive screen indicating clinician concern about possible cognitive frailty (eg because of Parkinson's disease, stroke, multiple sclerosis, alcohol excess etc). Pseudonymised data from previous relevant OUHFT-approved audits conducted prior to the implementation of EPR are also included, for example to establish the prevalence of delirium and cognitive impairment using different cognitive tests in consecutive admissions to acute general medicine.[1,2]

Out-patients

In addition, relevant OUHFT-approved audit data may be included on out-patients (including patients aged<65 years) deemed to be at-risk of cognitive or physical frailty as indicated by referral to vascular specialty clinics (eg TIA/minor stroke, peripheral vascular disease), memory clinics or geriatric medicine clinics where multimorbidity (the presence of two or more long-term conditions) is common. Manual extraction of audit data by the usual care team is necessary since at present, EPR recording of out-patient assessments is generally in the form of free text powernotes rather than structured data.

EPR Powerforms

EPR powerforms are bespoke electronic proformas "built" by the OUHFT EPR team and designed to capture structured clinical data. The cognitive screening form is an example of a powerform (see supplemental figure). The data entered into such a powerform can be easily extracted and analysed including all the individual powerform items (eg the individual AMTS questions). The structured format of powerforms also allows the information contained within to be displayed automatically in different locations in EPR. For example, the cognitive screening powerform results populate the observations tab and the "cognitive assessments" tab where all the cognitive screening results over time and across different encounters are shown allowing clinicians to see the cognitive trajectory. This tab also displays the clinical frailty score assuming the relevant powerform has been completed, so that the cognitive

For peer review only

assessments are put in the context of a global frailty assessment. The structured powerform data can also be included in algorithms for example, the AMTS score or untestability is included in the delirium susceptibility score algorithm which is calculated automatically within EPR and displayed in real time.[3]

EPR Powernotes

EPR powernotes are created by clinicians as electronic free text entries and are therefore entirely unstructured. Such free text data cannot be easily extracted or analysed and require the use of natural language processing algorithms. Information entered into a free text powernote, for example the AMTS score or documentation of delirium diagnosis, cannot therefore be automatically displayed in other parts or EPR or used in algorithms and quickly becomes "lost" amongst the mass of other entries.

References

1. Pendlebury ST, Lovett N, Smith S, Dutta N, Bendon C, Lloyd-Lavery A, et al. Observational, longitudinal study of delirium in consecutive unselected acute medical admissions: age-specific rates and associated factors, mortality and re-admission. *BMJ Open* 2015;5(11):e007808-e007808.

2. Pendlebury ST, Klaus SP, Mather M, de Brito M, Wharton RM. Routine cognitive screening in older patients admitted to acute medicine: abbreviated mental test score (AMTS) and subjective memory complaint versus Montreal Cognitive Assessment and IQCODE. *Age Ageing* 2015;44:1000-1005.

3. Pendlebury ST, Lovett NG, Smith SC, Wharton R, Rothwell PM. Delirium risk stratification in consecutive unselected admissions to acute medicine: validation of a susceptibility score based on factors identified externally in pooled data for use at entry to the acute care pathway. *Age Ageing* 2017;46:226-231.

For peer review only

Supplemental Figure

Supplementary Figure. The OUHFT cognitive screening EPR powerform including the 10-point AMTS, a brief screen for cognitive problems with a drop down list for reason for lack of completion (e.g. patient too unwell, aphasia); recording of formal dementia diagnosis (recorded as yes/no/uncertain); and recording of delirium diagnosis (recorded as yes/no/uncertain) informed by the CAM.

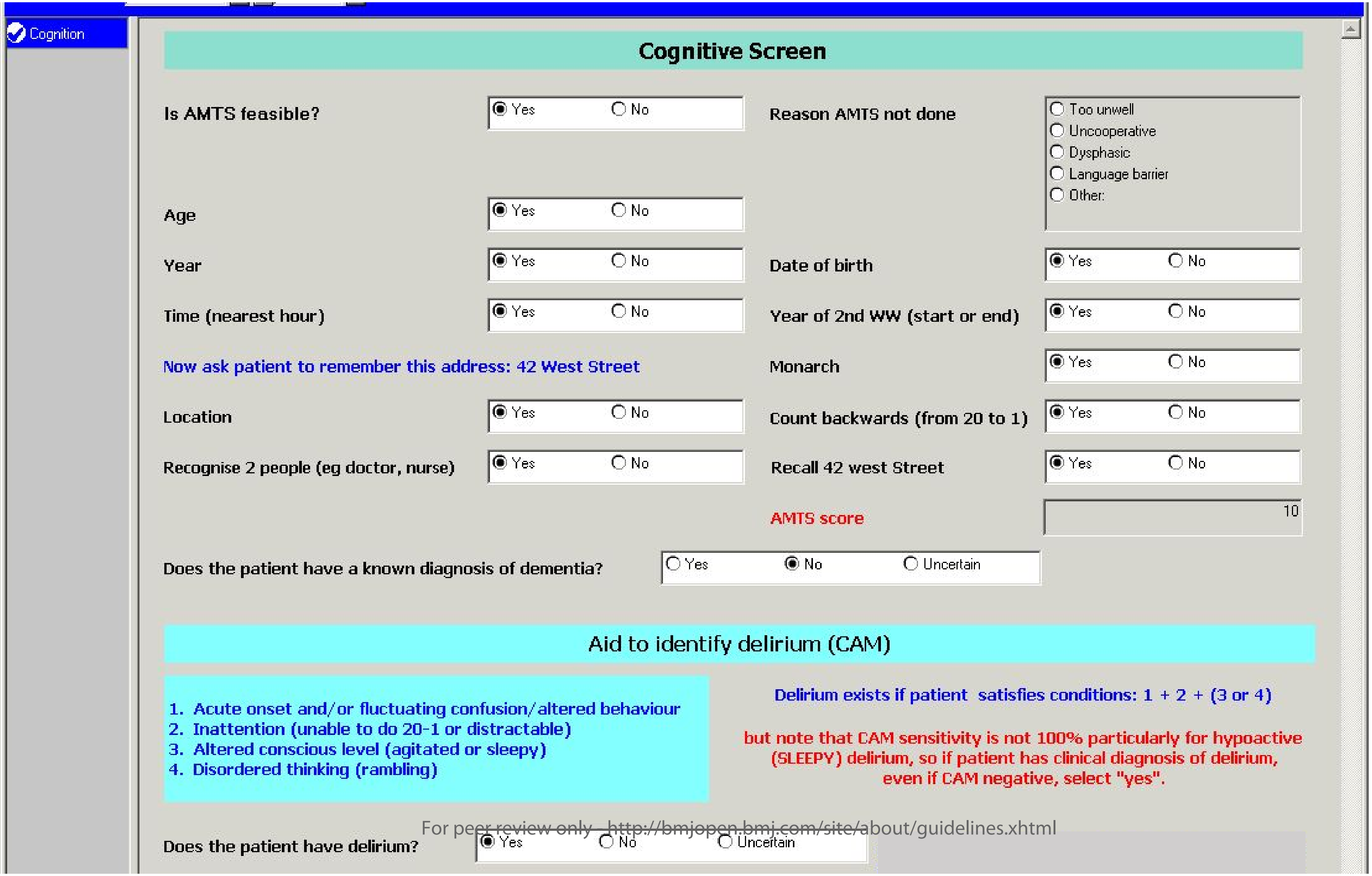

Cognition

Cognitive Screen

Is AMTS feasible? (•) Yes ( ) No

Reason AMTS not done: ( ) Too unwell ( ) Uncooperative ( ) Dysphasic ( ) Language barrier ( ) Other:

Age (•) Yes ( ) No

Year (•) Yes ( ) No

Date of birth (•) Yes ( ) No

Time (nearest hour) (•) Yes ( ) No

Year of 2nd WW (start or end) (•) Yes ( ) No

Now ask patient to remember this address: 42 West Street

Monarch (•) Yes ( ) No

Location (•) Yes ( ) No

Count backwards (from 20 to 1) (•) Yes ( ) No

Recognise 2 people (eg doctor, nurse) (•) Yes ( ) No

Recall 42 west Street (•) Yes ( ) No

AMTS score 10

Does the patient have a known diagnosis of dementia? ( ) Yes (•) No ( ) Uncertain

Aid to identify delirium (CAM)

1. Acute onset and/or fluctuating confusion/altered behaviour
2. Inattention (unable to do 20-1 or distractable)
3. Altered conscious level (agitated or sleepy)
4. Disordered thinking (rambling)

Delirium exists if patient satisfies conditions: 1 + 2 + (3 or 4)

but note that CAM sensitivity is not 100% particularly for hypoactive (SLEEPY) delirium, so if patient has clinical diagnosis of delirium, even if CAM negative, select "yes".

Does the patient have delirium? (•) Yes ( ) No ( ) Uncertain

For peer review only - http://bmjopen.bmj.com/site/about/guidelines.xhtml
